# Gesture deficits in psychosis and the combination of group psychotherapy and transcranial magnetic stimulation: A randomized clinical trial

**DOI:** 10.1038/s41380-025-03303-7

**Published:** 2025-10-08

**Authors:** Sebastian Walther, Lydia Maderthaner, Victoria Chapellier, Sofie von Känel, Daniel R. Müller, Stephan Bohlhalter, Mischa Baer, Anastasia Pavlidou

**Affiliations:** 1 University Hospital of Psychiatry and Psychotherapy Bern, Translational Research Center, University of Bern, Bern, Switzerland; 2 Translational Imaging Center (TIC), Swiss Institute for Translational and Entrepreneurial Medicine, Bern, Bern, Switzerland; 3 Department of Psychiatry, Psychosomatics, and Psychotherapy, Center of Mental Health, University Hospital of Würzburg, Würzburg, Germany; 4 Department of Basic and Clinical Neuroscience, Institute of Psychiatry, Psychology and Neuroscience, King’s College London, London, United Kingdom; 5 Graduate School for Health Sciences, University of Bern, Bern, Switzerland; 6 Department of Health Sciences and Medicine, University of Lucerne, Lucerne, Switzerland; 7 Department of Neurology, University of Zurich, Zurich, Switzerland; 8 Neurocenter, Cantonal Hospital of Lucerne, Lucerne, Switzerland; 9 Centre for Addictive Disorders, Department of Psychiatry, Psychotherapy and Psychosomatics, Psychiatric Hospital, University of Zurich, Zurich, Switzerland

**Keywords:** Schizophrenia, Neuroscience

## Abstract

Hand gesture deficits are common in schizophrenia predicting poor social functioning with no treatment currently available. We used 10-sessions of repetitive transcranial magnetic stimulation (rTMS; 2-weeks) over the right inferior parietal lobe (IPL) in combination with 16-sessions of social cognitive remediation group therapy (SCRT; 8-weeks) to examine improvements in hand gesture performance in schizophrenia. In this 3-arm, double-blind, randomized, sham-controlled trial, 89 patients were randomized and 73 received at least one session of rTMS/SCRT: 19 patients were allocated to the real rTMS + real SCRT treatment, 26 to the sham rTMS + real SCRT treatment, and 28 to sham SCRT treatment. Hand gesture performance along with socio-cognitive and functional assessments were examined at 2-weeks, 8-weeks, and 32-weeks follow-up. Of 73 patients analyzed, (57% male), 53 completed the intervention and week-8 assessments. At week-8 no difference in overall hand gesture performance accuracy was observed across treatments. However, at week-32 follow-up the real rTMS + real group SCRT treatment showed significant improvements in novel unlearned gestures (F_(6, 210)_ = 2.2; p-value = 0.04), and significant gains in social functioning/personal performance at week-8 and sustained at week-32 follow-up (all F-values > 2.6; all p-values < 0.05). No treatment effects were found for overall hand gesture performance accuracy. However, improvements in secondary outcomes such as novel unlearned gestures and social/personal functioning hold promise for testing optimized rTMS + group SCRT combinations. Future studies should explore the neural effects of rTMS over right IPL + group SCRT.

## Introduction

Schizophrenia is a severe mental-illness with multiple clinical characteristics including impairments in social-cognitive abilities, which greatly affect overall functioning and quality of life [1]. Central to the social challenges patients with schizophrenia encounter, is nonverbal communication and in particular gestures [2–4]. Hand gestures are movements used alone or in conjunction with speech to convey a meaning or idea [5]. Not only do patients with schizophrenia use fewer gestures during social interactions [6, 7], their performance accuracy of hand gestures is subpar [8–10]. This is true for tool-based, communicative, and novel meaningless gestures, with the deficit being more pronounced in the pantomime meaningless gestures [9, 10], all of which predict poor social and occupational functioning [11]. Such impairments also affect nonverbal cue interpretation and body-knowledge, indicating a broader communication deficit with no specific treatment [8].

Correct performance of gestures is highly dependent on the interplay of parietal, motor, and language areas of the praxis network. In schizophrenia, the functional and structural integrity of the praxis network is compromised [4, 12–16]. For example, altered activation in the left inferior parietal lobe (IPL) [17] was observed during an imitation finger-task [13], while planning of gestures showed neural alterations in the left inferior frontal gyrus (IFG) [18], supplementary motor area and IPL [12]. Therefore, neural modulation of key areas of the praxis network might be beneficial in improving gesture performance in schizophrenia. Early evidence suggests that a single-session of transcranial direct-current stimulation (tDCS) over the left frontal lobe enhances gesture interpretation, while a single-session of repetitive transcranial magnetic stimulation [19] over the right IPL improves accuracy [18, 20].

Varying rTMS protocols affect neural activity differently. For example, continuous theta burst stimulation [cTBS] is inhibitory while intermittent theta burst stimulation ([iTBS] is facilitatory [21], and induce enduring neural changes through long-term potentiation/depression that may elicit permanent and specific changes in neural circuits. In fact, evidence from previous studies show that rTMS improves symptom severity in depression and schizophrenia [22–25], including psychomotor slowing [26] with effects lasting weeks or even months post-treatment. Specific to gesture abilities, our previous single-session randomized, double-blind, sham-controlled trial found that a single cTBS session on the right IPL may enhance gesture accuracy in schizophrenia by inhibiting interhemispheric rivalry [20]. To explore this positive effect further we aimed at testing this treatment with repeated administration of cTBS on right IPL in 10-sessions over 2-weeks. Additionally, we included 16-sessions over 8-weeks of a tailored integrative broad-based social-cognitive remediation group therapy [SCRT [27, 28]]. It combines social and neurocognitive domains and is an already well-established treatment for social impairments and community functioning in schizophrenia [29–32]. A combined rTMS and SCRT approach may enhance outcomes in schizophrenia by concurrently targeting both neural circuits of the praxis network and social-cognitive processes. While rTMS modulates brain dysfunction, SCRT reshapes maladaptive thoughts and behaviors through social learning and support, potentially boosting neuroplasticity [33]. Testing real SCRT across both real and sham TMS groups clarifies the added value of TMS, while a sham SCRT group helps isolating the effects of each intervention and their interaction. Growing evidence supports the synergistic benefits of combining non-invasive brain stimulation (NIBS), such as rTMS and tDCS, with therapeutic interventions across several neuropsychiatric disorders [34]. In depression, studies show that pairing rTMS or tDCS with cognitive behavioral therapy or cognitive control training may enhance symptom reduction and may accelerate remission, particularly when stimulation coincides with task engagement [17, 19, 35–38]. In obsessive compulsive disorders, rTMS paired with exposure therapy or cognitive treatment significantly improved obsessive-compulsive symptoms and insight [35, 39], while in PTSD combining rTMS with trauma-focused exposure can modulate fear-related circuits and enhance symptom relief, and improve hyperarousal [40, 41]. Together, these findings highlight the potential of NIBS to improve the efficacy of psychological therapies by targeting and modulating relevant neural circuits during active cognitive engagement.

To this end, the current clinical trial includes a comprehensive social-cognitive behavioral battery, one group with both rTMS and group SCRT treatments, one group with sham rTMS and group SCRT treatment, and one group with no rTMS and sham SCRT treatment to disentangle unspecific effects of add on rTMS and group SCRT treatment from group SCRT treatment alone or from treatment as usual (sham group SCRT). We expect that repetitive rTMS sessions modulating IPL neural activity will enhance training effects during the SCRT group sessions, and thus hypothesize that this combination will be superior to group SCRT treatment alone, or treatment as usual (sham SCRT) in improving gesture performance accuracy (primary outcome). In addition, we expect these improvements to be accompanied by gains in social functioning and social cognition (secondary outcomes). This is based on evidence linking gesture performance accuracy to social cognitive functioning, suggesting that enhancing gesture skills may support more effective nonverbal communication and interpretation of social cues [8, 11].

## Methods

### Trial design

This 3-arm double-blind randomized, sham-controlled clinical trial of add-on rTMS and group SCRT took place at the University Hospital of Psychiatry and Psychotherapy in Bern Switzerland [42]. Sample-size estimation can be found in SI 1 in Supplement 2. No significant changes to the study protocol were made after the trial commenced. The trial was registered on September 17, 2019 at clinical trials.gov (NCT04106427) before any patients were enrolled (SI 2).

### Ethics approval and consent to participate

The study protocol (Supplement 1) was approved by the ethics committee of the canton of Bern (BASEC 2019-00798) and adhered to the Declaration of Helsinki. Written informed consent was obtained from all participants. The study methods adhered to the relevant guidelines.

### Participants

**Fig. 1:**
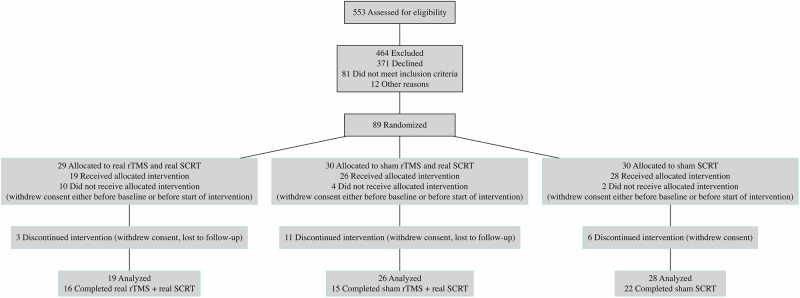
CONSORT FLOW DIAGRAM. Numbers for dropout reasons were summarized within groups to ensure data privacy. rTMS repetitive transcranial magnetic stimulation, SCRT social cognitive remediation therapy.

From October 30, 2019 to February 15, 2024, we screened 553 patients for eligibility, of which 89 were randomized: 29 to real rTMS and real SCRT, 30 to sham rTMS and real SCRT and 30 to sham SCRT (Fig. 1). We included patients aged between 18–65 years who were diagnosed with schizophrenia spectrum disorders according to DSM-5. Exclusion criteria included substance abuse (exception of nicotine), neurological conditions, epilepsy, head trauma, hearing problems, current pregnancy/breastfeeding, and no TMS/SCRT treatment in the past 3 months or 2 years respectively. The full inclusion/exclusion criteria can be found in Supplementary Table 1 in Supplement 2. Throughout the trial patients continued preexisting medication including antipsychotics and antidepressants. A total of 73 patients (59 patients with schizophrenia (81%) and 14 (19%) with schizoaffective disorder) received at least one rTMS/group SCRT session (modified intention-to-treat [ITT] group), however the number of completed assessments during follow-up varied. Follow-up was completed on April 24, 2024.

### TMS Protocol

Stimulation was delivered using the MagPro x100 or MagPro R30 with theta burst option. Both are manufactured by Tonica Elektronik and dispersed by MagVenture. We used the MCF-B70 coil to deliver real TMS stimulation and the MCF-P-B65 to deliver sham stimulation. Application of rTMS protocols followed published guidelines [43, 44]. Both real and sham protocols were delivered in 10 daily sessions over 2-weeks targeting the right IPL located at P4/CP4 of the EEG 10–20 system. For the patients who underwent MRI (n = 20; 45%) we used the Brainsight Neuronavigation System dispersed by BrainBox Ltd to locate the right IPL using their structural T1-image. The coils and neuronavigation system used, as well as the duration for both real and sham protocols were identical (SI 3 in Supplement 2).

Two-sessions of cTBS included 801 pulses (1602 total) in 267 bursts at an intensity of 100% resting motor-thresholds (44-second duration each session). Each session was separated with a 15-minute break. This protocol was similar to our previous study [20]. For sham stimulation, the protocol matched the cTBS protocol, but a placebo coil was used, mimicking the real coil in sound and appearance without magnetic emissions.

### SCRT Protocol

Group therapy was delivered using the Integrated Neurocognitive Therapy (INT) approach [28], in accordance to the MATRICS initiative [45], which includes modules tailored in improving both social cognition and neurocognition in patients with schizophrenia [2, 31, 33, 46–48]. For the real SCRT group only MATRICS dimensions relevant to gesture production were included, and the education-compensation-transfer process was shortened due to limited time (SI 4, Supplementary Table 2a in Supplement 2). In contrast, sham SCRT group (SI 4, Supplementary Table 2b in Supplement 2) primarily focused on psychoeducation, information on diet and sleep hygiene, exercise, stress-free environment, and the arts, while engaging them in leisure activities such as mindfulness, walking, and visits to museums. Patients in the sham group SCRT benefited from an interactive environment, but without the add on social-cognitive training. Both real and sham group SCRT were delivered biweekly for 8-weeks, totaling 16 sessions. Each session lasted for 90-minutes and was led by a head-therapist (V.C.) and a co-therapist both supervised by an INT-expert (D.M.).

### Outcomes

The primary outcome was change in gesture performance accuracy following interventions over timepoints (Baseline, Week-2, and Week-8). We also investigated if any changes occurred or remained at Week-32 follow-up. Gesture performance accuracy was measured using the Test of Upper-Limb Apraxia (TULIA) [49] which contains two domains and three categories of gestures. TULIA is videorecorded and assessed by an independent examiner blinded to the treatment arms. (SI 5 in Supplement 2).

Secondary outcomes included changes over timepoints (Baseline, Week-2, and Week-8) in social-cognition (Mini-Profile of Nonverbal Sensitivity [Mini-PONS] [50, 51]), gestural knowledge (Postural Knowledge Test [PKT] [52, 53]), managing emotions (Mayer-Salovey-Caruso Emotional Intelligence test [MSCEIT] [54]; SI 6 in Supplement 2) and expert ratings covering illness severity (Positive and Negative Symptom Scale [PANSS] [55]; Brief Negative Symptom Scale [BNSS [56]]), social functioning (Social and Occupational Functioning Assessment Scale [SOFAS [57]]; SI 6 in Supplement 2]; Specific Level of Functioning [SLOF [58]]; Personal and Social Performance [PSP [57]]; SI 6 in Supplement 2) and functional capacity (University of California San Diego Performance-Based Skills Assessment [UPSA brief] [59]); In addition, we collected self-reported gesture perception and production (Brief Assessment of Gestures [BAG [60]] and negative symptoms (Self-evaluation of Negative Symptoms [SNS] [61]). Similarly to our primary outcome analyses, we investigate if the changes observed after completion of the interventions occur or carry-over to Week-32 follow-up.

### Procedures

**Fig. 2:**
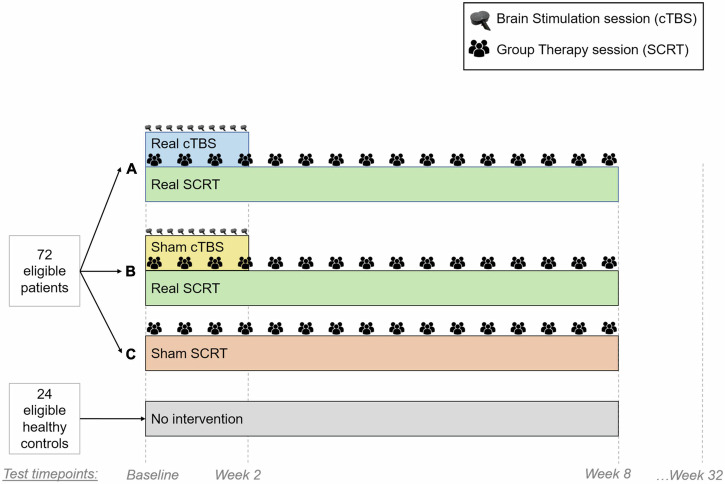
Illustration of the clinical trial setup. Reproduced from Chapellier et al. [42] “Brain Stimulation and Group Therapy to Improve Gesture and Social Skills in Schizophrenia-The Study Protocol of a Randomized, Sham-Controlled, Three-Arm, Double-Blind Trial”, Frontiers in Psychiatry, under the terms of the Creative Commons Attribution License (CC BY 4.0). Both real and sham cTBS treatment arms underwent 10 sessions of neurostimulation, while all treatment arms participated in 16 sessions of SCRT. cTBS continuous theta burst stimulation. SCRT social cognitive remediation therapy.

After providing informed consent and before any baseline assessments took place the real rTMS, SCRT, and sham groups were randomly allocated to treatment arms (Fig. 2). Safety outcomes included adverse stimulation effects after each rTMS session and after 10-sessions (Week-2). Adverse events and behavioral outcomes including experience and satisfaction were collected after 16 sessions (Week-8) of either real or sham SCRT (SI 9 in Supplement 2). At baseline, Week-2, Week-8, and Week-32 patients’ TULIA performance, social-cognition and symptom severity were measured; while managing emotions, social functioning and functional capacity were measured at Week-8 and Week-32. We summarized antipsychotic doses as mean olanzapine-equivalents (OLZ) [62].

### Randomization

Organizational restrictions prevented simultaneous real and sham SCRT. Therefore, we divided the real and sham SCRT into separate time-blocks. Patients were randomized 1:1 to 1 of the 2 treatments with real SCRT using a research randomizer online-tool in one time-block. We did this after recruiting 6–8 patients. In a different time-block, we assigned another 6–8 patients to the sham SCRT group (SI 7 in Supplement 2). A total of 14 time-blocks were administered; 9 blocks with real SCRT and 5 blocks with sham SCRT. The randomization lists generated were accessible to two people (S.W. and A.P.), and treatment allocation was communicated only to the person administering rTMS and SCRT interventions (V.C.).

### Blinding

Outcome assessors, clinicians, and patients were blinded to the treatment arms. Duration of treatment, setting, and TMS machinery were identical for all patients. In 53 patients, we assessed the suspected type of intervention (real or sham) received at Week-2.

### Statistical analyses

Primary and secondary outcomes were analyzed by 2 researchers (S.W. and A.P.) with RStudio version 2024.12.0 + 467 (R-Foundation for Statistical Computing, Vienna, Austria). All analyses were done in the modified ITT sample (n = 73 with at least 1 rTMS/SCRT session) using the last-observation-carried-forward (LOCF) method to account for missing data. We compared improvement of gesture performance accuracy over timepoints (Baseline to Week-8 and Baseline to Week-32) between treatment arms using repeated-measure ANOVAs for the total TULIA score, domains, and categories. PostHoc-tests were corrected for multiple comparisons using false discovery rate. P-values < 0.05 were considered significant. Similarly, to test the effects of rTMS and SCRT on secondary outcomes (i.e. BNSS, Mini-PONS, SOFAS,) we also used repeated-measure ANOVAs (P-values < 0.05 significant). Frequencies of adverse events, blinding evaluation, and satisfaction ratings were calculated using binomial logistic regression [63] (SI 8 in Supplement 2) and Kruskal-Wallis tests (P-values < 0.05 significant).

## Results

### Recruitment

Of the 73 patients analyzed, 57% were male and 43% were female, with a mean age of 40.1 years (±SEM 2.5 years). A total of 53 patients completed the intervention period, as well as, the week-8 assessments (16 in the real rTMS and real group SCRT, 15 in the sham rTMS and real group SCRT, and 22 in the sham group SCRT). Reasons for discontinuation were withdrawal of consent (n = 12) and lost to follow-up (n = 8; Fig. 1; Supplementary Table 4 in Supplement 2).

Baseline demographic and clinical characteristics are given in Table 1. No differences between treatment arms were observed. The percentage of patients who completed or attended treatments in each arm is given in Supplementary Table 3 in Supplement 2.

**Table 1:** Demographic, Clinical, and Outcome Baseline Characteristics of ITT.

	Mean ± SEM			
Characteristics	real rTMS + real SCRT (n = 19)	sham rTMS + real SCRT (n = 26)	Sham SCRT (n = 28)	Kruskal-Wallis
**Age (years)**	42.2 ± 2.7	40.1 ± 1.9	37.4 ± 2.3	X^2^ = 1.8; p = 0.4
**Sex No. (%)**				X^2^ = 2.9; p = 0.2
**Female**	35.0%	45.0%	57.0%	
**Male**	65.0%	55.0%	43.0%	
**Education (years)**	13.9 ± 0.6	12.9 ± 0.7	13.7 ± 0.7	X^2^ = 0.8; p = 0.6
**Duration of illness (years)**	13.8 ± 2.6	15.7 ± 2.6	14.7 ± 2.2	X^2^ = 4.5; p = 0.1
**Baseline mean OLZ-eq**	14.4 ± 2.7	10.8 ± 1.9	9.8 ± 1.5	X^2^ = 2.2; p = 0.3
**Outcomes**				
**TULIA total**	192.7 ± 3.9	191.1 ± 4.4	193.0 ± 3.0	X^2^ = 0.3; p = 0.8
**Imitation (TULIA)**	101.6 ± 1.7	100.1 ± 2.1	101.7 ± 1.3	X^2^ = 0.1; p = 0.9
**Pantomime (TULIA)**	91.1 ± 2.6	91.0 ± 2.8	91.2 ± 2.0	X^2^ = 0.3; p = 0.8
**Imitation Meaningless (TULIA)**	35.2 ± 0.6	34.6 ± 0.7	35.5 ± 0.6	X^2^ = 0.8; p = 0.6
**Imitation Intransitive (TULIA)**	35.4 ± 0.6	34.4 ± 0.8	34.8 ± 0.5	X^2^ = 0.4; p = 0.8
**Imitation Transitive (TULIA)**	31.0 ± 1.1	31.1 ± 0.9	31.4 ± 0.6	X^2^ = 0.0; p = 1
**Pantomime Meaningless (TULIA)**	30.9 ± 1.2	31.0 ± 1.4	32.1 ± 1.0	X^2^ = 0.7; p = 0.7
**Pantomime Intransitive (TULIA)**	31.1 ± 0.9	30.1 ± 0.9	30.5 ± 0.7	X^2^ = 0.3; p = 0.9
**Pantomime Transitive (TULIA)**	29.1 ± 1.3	29.9 ± 0.9	28.5 ± 0.9	X^2^ = 1.4; p = 0.5
**Mini-PONS Total**	42.4 ± 1.3	42.0 ± 1.1	43.5 ± 1.5	X^2^ = 1.8; p = 0.4
**Mini-PONS Face**	10.8 ± 0.4	11.1 ± 0.4	10.8 ± 0.4	X^2^ = 0.2; p = 0.9
**Mini- PONS Hands**	10.6 ± 0.5	9.8 ± 0.4	11.0 ± 0.5	X^2^ = 3.3; p = 0.2
**Mini- PONS Voice**	10.5 ± 0.5	9.8 ± 0.4	10.3 ± 0.4	X^2^ = 0.9; p = 0.6
**Mini- PONS Face** + **Voice**	10.4 ± 0.5	11.3 ± 0.4	11.5 ± 0.6	X^2^ = 4.2; p = 0.1
**PKT Total**	14.4 ± 0.9	14.5 ± 0.9	14.5 ± 0.6	X^2^ = 0.6; p = 0.7
**MSCEIT Managing Emotion**	86.0 ± 2.1	88.8 ± 1.9	84.4 ± 1.8	X^2^ = 1.9; p = 0.4
**MSCEIT Emotion Management**	87.4 ± 2.0	89.5 ± 1.7	86.7 ± 1.7	X^2^ = 1.5; p = 0.5
**MSCEIT Social Management**	87.1 ± 2.1	89.5 ± 1.9	85.0 ± 1.8	X^2^ = 2.6; p = 0.3
**PANSS Positive**	16.2 ± 1.7	12.6 ± 1.0	13.9 ± 0.8	X^2^ = 2.6; p = 0.3
**PANSS Negative**	20.1 ± 2.0	17.7 ± 1.7	15.2 ± 1.2	X^2^ = 3.3; p = 0.2
**PANSS Total**	72.2 ± 6.0	60.8 ± 3.9	60.0 ± 2.7	X^2^ = 3.3; p = 0.2
**BNSS Total**	33.9 ± 3.9	27.8 ± 3.2	26.7 ± 3.2	X^2^ = 2.2; p = 0.3
**SNS Total**	14.6 ± 1.5	15.8 ± 1.8	16.7 ± 1.2	X^2^ = 0.9; p = 0.7
**BAG mean Total**	3.1 ± 0.1	3.1 ± 0.1	3.1 ± 0.1	X^2^ = 0.3; p = 0.9
**SOFAS Total**	44.4 ± 2.4	51.6 ± 2.5	47.9 ± 1.8	X^2^ = 3.4; p = 0.2
**SLOF Total**	174.9 ± 5.8	183.7 ± 3.5	179.1 ± 3.9	X^2^ = 1.3; p = 0.5
**PSP Total**	46.1 ± 2.7	51.4 ± 2.5	47.1 ± 2.0	X^2^ = 2.2; p = 0.3
**UPSA-B Total**	74.4 ± 6.0	76.3 ± 4.2	79.6 ± 2.2	X^2^ = 0.1; p = 0.9

*BAG* brief assessment of gestures, *BNSS*, brief negative symptom scale, *Mini-PONS* mini profile of nonverbal sensitivity, *MSCEIT* mayer-salovey-caruso emotional intelligence test, *OLZ-eq*, olanzapine equivalent, *PANSS* positive and negative symptom scale, *PKT* postural knowledge test, *PSP* personal and social performance, *rTMS* repetitive transcranial magnetic stimulation, *SCRT* social cognitive remediation therapy, *SLOF* specific level of functioning, *SNS* self-evaluation of negative symptoms, *SOFAS* social and occupational functioning assessment scale, *TULIA* test of upper limb apraxia, *UPSA-B* university of california san diego performance-based skills assessment brief.

### Primary outcome

**Fig. 3:**
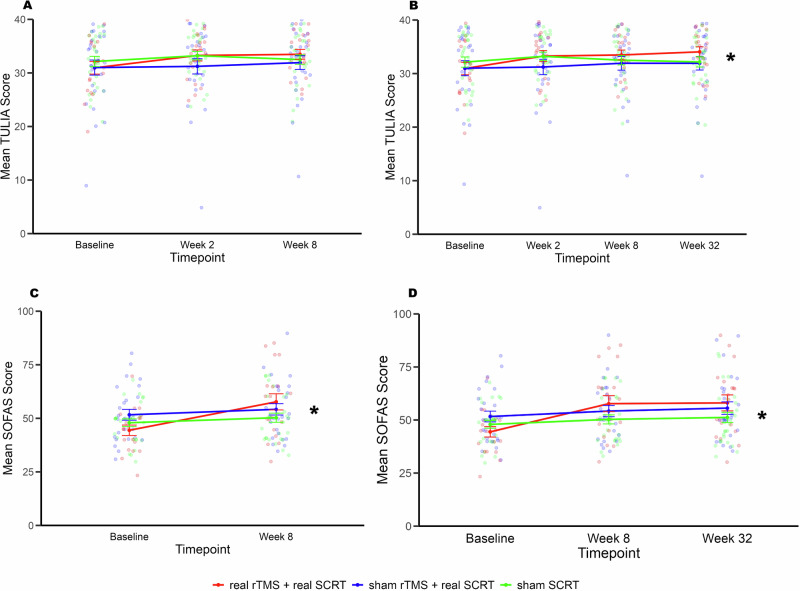
Course of Pantomime Meaningless gesture category and Social Functioning. The line plots depict performance across timepoints for real rTMS + real SCRT (red), sham rTMS and real SCRT (blue) and sham SCRT (green). **A** shows performance of pantomime meaningless gestures from Baseline to Week-8. **B** shows performance of pantomime meaningless gestures from Baseline to Week-32. **C** shows social functioning performance from Baseline to Week-8. **D** shows social functioning performance from Baseline to Week-32. Analyses was done using last observation carried forward to account for missing values. Error bars represent SEMs. rTMS indicates repetitive transcranial magnetic stimulation; SCRT indicates social cognitive remediation therapy. ^*^Significant effect at *P*  <  0.05 for real rTMS + real SCRT from baseline.

In the ITT analyses with the LOCF, repeated-measures ANOVAs from Baseline to Week-8 comparing the 3 treatment arms revealed significant effects of time in gesture performance accuracy in the total TULIA score, pantomime domain score as well as, in the pantomime meaningless and intransitive categories (all F_(2140)_ > 4.3; p-value < 0.01; Supplementary Table 5, Fig. 3A). A similar pattern of results was observed for Baseline to Week-32, with the exception of the pantomime meaningless category, where a significant effect of time-by-treatment arm was observed (F_(6, 210)_ = 2.2; p-value = 0.04; η^2^p = 0.06; Supplementary Table 6; Fig. 3B). Posthoc comparisons revealed an improvement in performance accuracy of pantomime meaningless gestures only for the real rTMS and real group SCRT treatment arm (Supplementary Table 6; Fig. 3B).

### Secondary outcomes

Repeated-measures ANOVAs of the secondary outcomes from Baseline to Week-8 revealed significant effects of time in PANSS positive, negative, total, BNSS Total, SOFAS, SLOF and PSP, while time-by-treatment arm interaction was exclusive to SOFAS (F_(2, 70)_ = 6.2, p-value = 0.003; η^2^p = 0.15; eTable 5; Fig. 3C) and PSP (F_(2, 70)_ = 3.5, p-value = 0.03; η^2^p = 0.09; Supplementary Table 5; Supplementary Figure 1). Posthoc comparisons revealed a pronounced improvement in social and occupational functioning only for the real rTMS and real group SCRT treatment arm. A similar pattern of results was observed for Baseline to Week-32 with an additional significant effect of time observed on UPSA-B (Supplementary Table 5). The time-by-treatment arm interaction on SOFAS (F_(4, 140)_ = 4.2, p-value = 0.003; η^2^p = 0.11; Supplementary Table 6; Fig. 3D) and PSP (F_(2, 70)_ = 2.6, p-value = 0.04; η^2^p = 0.07; Supplementary Table 5; Supplementary Figure 2) remained with real rTMS and real group SCRT again showing the most prominent improvement that endured at Week-32 follow-up.

### Blinding efficacy

Patients receiving rTMS and group SCRT interventions were unable to identify their assigned treatment (X^2^ = 2.4, p-value = 0.3). Twenty-three patients (43%) correctly guessed that they received real or sham rTMS/SCRT.

### Adverse events

Approximately half of the participants experienced adverse events at least once with no significant differences between treatment arms at any timepoint (all p-values > 0.09; Table 2a-c; SI 10 and Supplementary Tables 7-8 Supplement 2). Two serious adverse events occurred—one in the sham rTMS and real SCRT arm (severe headache and vomiting) and one in the sham SCRT arm (hospitalization due to psychotic relapse)—both determined to be unrelated to the treatment.

**Table 2:** a Adverse events per rTMS session for each arm. b Adverse events after 2 weeks of rTMS treatment for each arm.. c Adverse events after 8 weeks of rTMS treatment for each arm.

a								
	N (%)							
Session	Dizziness	Dizziness	Fatigue	Fatigue	Headache/Neck pain	Headache/Neck pain	Other^a^	Other^b^
	real rTMS + real SCRT (n = 19)	sham rTMS + real SCRT (n = 26)	real rTMS + real SCRT (n = 19)	sham rTMS + real SCRT (n = 26)	real rTMS + real SCRT (n = 19)	sham rTMS + real SCRT (n = 26)	real rTMS + real SCRT (n = 19)	sham rTMS + real SCRT (n = 26)
Session 1	2 (10.5%)	1 (3.8%)	5 (26.3%)	2 (7.7%)	2 (10.5%)	2 (7.7%)	12 (63.2%)	12 (46.2%)
Session 2	0	0	1 (5.3%)	0	5 (26.3%)	6 (23.1%)	11 (57.9%)	17 (65.4%)
Session 3	0	0	0	2 (7.7%)	3 (15.8%)	5 (19.2%)	13 (68.4%)	13 (50%)
Session 4	2 (10.5%)	0	1 (5.3%)	1 (3.8%)	4 (21.1%)	5 (19.2%)	12 (63.2%)	13 (50%)
Session 5	2 (10.5%)	0	1 (5.3%)	0	5 (26.3%)	2 (7.7%)	11 (57.9%)	11 (42.3%)
Session 6	2 (10.5%)	0	1 (5.3%)	1 (3.8%)	4 (21.1%)	4 (15.4%)	12 (63.2%)	12 (46.2%)
Session 7	0	1 (3.8%)	0	1 (3.8%)	2 (10.5%)	3 (11.5%)	10 (52.6%)	13 (50%)
Session 8	2 (10.5%)	1 (3.8%)	1 (5.3%)	2 (7.7%)	3 (15.8%)	3 (11.5%)	10 (52.6%)	10 (38.4%)
Session 9	1 (5.3%)	0	1 (5.3%)	1 (3.8%)	1 (5.3%)	4 (15.4%)	11 (57.9%)	12 (46.2%)
Session 10	1 (5.3%)	0	0	1 (3.8%)	2 (10.5%)	2 (7.7%)	11 (57.9%)	14 (53.8%)

N (%)		
Variable	real rTMS + real SCRT (n = 19)	sham rTMS + real SCRT (n = 26)
b		
**No adverse effects**	12 (63.2%)	11 (42.3%)
**Dizziness**	1 (5.3%)	2 (7.7%)
**Fatigue**	1 (5.3%)	3 (11.5%)
**Headache/Neck Pain**	5 (26.3%)	9 (34.6%)
**Other**	2 (10.5%)^a^	7 (26.9%)^b^

c		
**No adverse effects**	13 (68.4%)	15 (57.7%)
**Dizziness**	1 (5.2%)	2 (7.7%)
**Fatigue**	1 (5.2%)	1 (3.8%)
**Headache/Neck Pain**	6 (31.6%)	6 (32.1%)
**Other**	1 (5.2%)^a^	3 (11.5%)^b^

Some patients experienced more than one side effect.

some patients experienced more than one adverse event.

*rTMS* repetitive transcranial magnetic stimulation, *SCRT* social cognitive remediation therapy.

^a^Including light pulsating, tingling or burning sensation, light flickering, burning sensation or jaw tension.

^a^Including light pulsating, tingling, or burning sensation.

^b^Including pressure on the head, tingling, eye blinking and flickering, increased anxiety, or bizarre thoughts and sensations.

^b^Including pressure on the head, eye blinking, and increased sensations.

## Discussion

Impairments in hand gesture performance occur frequently in schizophrenia and are associated with poor community functioning [4, 8, 11] for which no treatment is currently available. Neuroimaging research linked deficits in hand gesture performance in schizophrenia to altered functional and structural activity/connectivity of the praxis network, which comprises of parietal, motor and language areas [12, 15, 16]. This double-blind randomized sham-controlled clinical trial tested whether the combination of 10 sessions of add-on rTMS on the right IPL and 16 sessions of add-on group SCRT treatment would improve hand gesture performance in schizophrenia across different domains and categories [42]. Contrary to our hypothesis, add-on real rTMS and real SCRT did not improve overall hand gesture performance. However, we observed improvement of hand gesture performance in the pantomime meaningless category, when including Week-32 follow-up, and improvements in personal and social performance and functioning during Week-8 and Week-32 follow-up exclusively in the real rTMS and real SCRT treatment arm.

This result is in contrast to our previous clinical trial, which demonstrated immediate improvement in hand gesture performance following a single-rTMS session on right IPL [20]. Our previous trial aimed to assess the efficacy of different rTMS protocols on the praxis network. It did not include repeated rTMS, add-on SCRT treatment, or an analysis of specific hand gesture categories. However, the results from the current clinical trial reveal a more nuanced picture. In particular, we found unspecific time-effects across treatment arms, as all patients improved in symptom severity including negative symptoms, and overall hand gesture performance, in particular, pantomime gestures including the intransitive gesture category, which are highly-communicative gestures. Improved pantomime gestures may have meaningful clinical and functional implications. In daily life, pantomime gestures play a key role in nonverbal communication, particularly in situations where speech is limited (e.g., noisy environments or language barriers). Although assessed in TULIA through verbal command, these gestures, especially intransitive ones closely resemble spontaneous gestures used in real-world interactions. Because pantomime gestures engage motor planning, semantic knowledge, and executive functions, they reflect broader praxis abilities [64]. Their production relies on neural systems implicated in action understanding and planning such as IPL, IFG, and premotor cortex. Notably, impairments in both pantomime and spontaneous gestures are common in schizophrenia and are linked to poorer social functioning [8, 12, 65]. Thus, enhancing pantomime gestures may support better social communication and functional outcomes. From the current trial, it seems that the general group setting with biweekly sessions has strong benefits for schizophrenia. However, SCRT covered only part of integrated neurocognitive therapy (INT); key domains and session-time were reduced, possibly contributing to less consistent effects on social cognition and symptoms. As a trade-off, the sham SCRT arm proved to be a highly active control condition, far more effective than waiting list, often applied in psychological treatment trials [66–68]. The sham SCRT group participated in mindfulness and psychoeducation activities to maintain blinding while fostering a communal experience, reducing stress, enhancing comfort, and promoting well-being through self-awareness and presence [69, 70]. As such the group setting may have supported patients’ social-cognitive and emotional regulation indirectly, by fostering attentional focus, psychological containment, and a sense of shared purpose, even in the absence of explicit focused intervention.

We observed no specific time or time-by-treatment effects in imitative, or tool-based gestures, as well as social cognition or postural knowledge. While we could argue that other areas of the praxis network (i.e. IFG) in combination with our tailored group SCRT treatment might be more ideal for these specific outcomes, it might also be explained by insufficient statistical power. The sample size for all groups, especially for the real rTMS and real group SCRT treatment arm was suboptimal and the LOCF method is very conservative.

Notably, we observed beneficial time-by-treatment effects exclusively for pantomime meaningless gestures with real rTMS on right IPL and real group SCRT. The IPL is a key node in the brain’s praxis network, crucial for planning and executing gestures. In schizophrenia, gesture impairments are linked to grey matter loss in the IPL and connected regions, as well as disrupted network efficiency and white matter integrity [14, 71]. Similar patterns appear in stroke and Parkinson’s disease [72, 73]. cTBS to the right IPL was shown to improve gesture performance, suggesting that modulating overactive areas may rebalance network function [20]. This supports theories of state-dependent neuromodulation, where stimulation enhances learning by shifting brain state [74]. cTBS over the right IPL may facilitate left-hemispheric praxis networks via transcallosal disinhibition, improving gesturing in healthy and stroke populations [75, 76]. fMRI in Parkinson’s disease further links fine motor deficits to overactivation of the left praxis network and compensatory recruitment of temporal motor memory areas [77]. In this framework cTBS suppress maladaptive IPL activity and thereby enhance the effectiveness of SCRT. Future fMRI and connectivity studies could further clarify how IPLtargeted interventions support recovery in action planning networks.

Pantomime meaningless gestures are novel, unlearned gestures that are not tied to pre-existing motor patterns and external cues (i.e., imitation) and do not depend on semantic and symbolic processes [78, 79]. The improvement of pantomime meaningless gestures suggests the combination with rTMS and SCRT enhances cognitive flexibility, motor planning, and spatial awareness all of which are required to successfully execute novel, unlearned gestures [80–82]. The lack of significant improvement in transitive and intransitive gestures after combined rTMS and SCRT may reflect the complex cognitive and neural demands of these gestures. Unlike meaningless gestures, they rely on symbolic and semantic processing, engaging a broader network beyond the IPL, including the left IFG and temporal cortex [83, 84]. Stimulating the IPL alone may not sufficiently impact this network. These gesture types require integration across semantic and motor pathways, and deficits may persist unless regions like the IFG and middle temporal gyrus are also targeted [84, 85]. Additionally, SCRT may not have adequately addressed the symbolic aspects of gesture use. Future interventions might benefit from multisite neuromodulation or combining stimulation with semantic-action training to more directly support these processes. Alongside improvements in meaningless pantomime performance, we observed gains in personal and social functioning at Week-8 that were sustained through Week-32 (PSP, SOFAS), with similar trends in functional capacity (UPSA). This suggests a generalized carry-over effect of nonverbal communication skills transfer to general social functioning, improving patients’ ability to navigate novel cues [86].

Both rTMS and SCRT treatments where well tolerated. Two serious adverse events occurred but were unrelated to the study procedures. Mild and transient adverse events were noted in all treatment arms.

Ideally, this study should be replicated in larger multicenter trials. Both SCRT content and rTMS protocols can be readily optimized to enhance effects on social-cognitive skills, for example with accelerated rTMS [87] or more targeted training-sessions [88]. Other praxis network targets using non-invasive brain stimulation with SCRT could be tested including transdiagnostic studies [89–93]. Moreover, future studies should consider rTMS in combination with virtual reality; a method that provides a highly controlled, immersive and interactive environment that simulates complex real-life scenarios could be explored [94–96]. Further, to better connect neural and behavioral changes with real-world functioning, future research should include outcomes like employment, social engagement, and interpersonal skills. Tools like ecological momentary assessment and digital phenotyping can capture real-time data on daily behaviors and emotions, helping to assess whether lab-based gains translate into everyday life [97, 98]. These methods can also reveal how neural states, cognitive training, and social functioning interact in natural settings, improving ecological validity and clinical impact.

### Limitations

Using a 3-arm design we tested the combined effects of repeated rTMS and group SCRT on hand gesture performance in schizophrenia. The study has some limitations. First, although OLZ was similar across treatment arms other medications (i.e. antidepressants) might affect the outcome. Second, while training effects from repeated outcome exposure are possible, we minimized this by randomizing trial order and allowing sufficient time between follow-ups. Third, randomization was conducted before any baseline assessments took place, thus the ITT population included all patients who received at least one rTMS/SCRT session, in line with other rTMS studies in psychiatry but different from drug trials [26, 99, 100]. Finally, 20 patients (27% of ITT) dropped out before week-8 assessments. LOCF analysis accounted for dropouts but introduced a pessimistic estimation of outcomes.

## Conclusions

In this randomized clinical trial, the combination of rTMS and SCRT did not improve overall hand gesture performance. However, improvements were observed in a specific category of gestures (pantomime meaningless), as well as social and personal functioning, suggesting important beneficial effects of the combined treatment. Both intervention components can be intensified in future trials with larger sample sizes.

## Supplementary information

Supplementary Material - methods and results

## Data Availability

The dataset analysed during the current study is not publicly available as participants did not consent to broad data sharing of their health records. But deidentified parts of the data may be provided by the corresponding author on reasonable request.
