# Supplementary material for: Gesture deficits in psychosis and the combination of group psychotherapy and transcranial magnetic stimulation: A randomized clinical trial
Source: Mol Psychiatry. 2025 Oct 8;30(12):5790–9. doi: 10.1038/s41380-025-03303-7 (PMC12602348; doi:10.1038/s41380-025-03303-7)
Supplement: Supplementary file 1 — Supplementary Material - methods and results [file 41380_2025_3303_MOESM1_ESM.docx]

SUPPLEMENTAL INFORMATION FOR

**Gesture deficits in psychosis and the combination of group psychotherapy and transcranial magnetic stimulation: A randomized clinical trial.**

Sebastian Walther, Lydia Maderthaner, Victoria Chapellier, Sofie von Känel, Daniel R. Müller, Stephan Bohlhalter, Mischa Baer, Anastasia Pavlidou

**Supplementary Methods**

**SI 1.** Sample size estimation

**SI 2.** Trial registration

**Supplementary Table 1.** Inclusion/Exclusion criteria

**SI 3.** TMS Machinery

**SI 4.** SCRT Material

**Supplementary Table 2a:** Real SCRT Modules

**Supplementary Table 2b:** Sham SCRT Sessions

**SI 5.** Primary outcome and subscores

**SI 6.** Secondary outcome and subscores

**SI 7.** Randomization and allocation concealment

**SI 8**: Binomial logistic regression

**SI 9.** Adverse events after SCRT treatment

**Supplementary Results**

**Supplementary Table 3:** Attendance per treatment arm

**Supplementary Table 4:** Dropout Reasons for ITT

**Supplementary Table 5:** Primary and secondary outcomes according to treatment arm for ITT with LOCF until Week-8

**Supplementary Table 6:** Primary and secondary outcomes according to treatment arm for ITT with LOCF until Week-32

**Supplementary Table 7**: Output for Binomial logistic regression for adverse events following rTMS

**SI 10:** Adverse Events

**Supplementary Table 8**: Adverse events after 8 weeks of SCRT treatment for the ITT group

**Supplementary Figure 1** Course of PSP until Week-8 for all treatment arms

**Supplementary Figure 2** Course of PSP until Week-32 for all treatment arms

Supplementary Methods

*SI 1: Sample size estimation*

The main effects of add on rTMS and SCRT interventions on gesture performance accuracy were calculated in a repeated measures ANOVA design where Groups (real rTMS and SCRT; sham rTMS and real SCRT; sham SCRT) and Timepoints (Baseline, Week-2, Week-8) as well as their interaction were added as factors. Assuming a medium effects size (f = 0.15) in a repeated measures ANOVA with moderate to high correlation between timepoints (0.75), a power of 0.95 and an alpha = 0.05 we needed 72 patients (24 per group)^1^.

*SI 2: Trial Registration*

The trial was first registered 3 months before the enrollment of the first patient. On January 7, 2020 the Ethics committee of the canton of Bern communicated final approval of the protocol. On January 13, 2020 the first patient provided written informed consent. Trial registration on clinicaltrials.org was conducted on September 17, 2019 before the trial commenced and no changes were made to the design after that.

*Supplementary Table 1: Inclusion/Exclusion criteria*

| **Detailed inclusion and exclusion criteria** | |
| --- | --- |
| **Inclusion** | - 18-65 years of age - Right-Handed - Schizophrenia spectrum disorders according to DSM-5 |
| **Exclusion** | - Substance abuse or dependence other than nicotine. - Past or current medical or neurological condition associated with impaired or aberrant movement, such as brain tumors, stroke, M. Parkinson, M. Huntington, dystonia, or severe head trauma with subsequent loss of consciousness. - Epilepsy or other convulsions. - History of any hearing problems or ringing in the ears. - Standard exclusion criteria for cerebral magnetic resonance imaging scanning and transcranial magnetic stimulation (TMS); e.g. metal implants, claustrophobia. - Any transcranial magnetic stimulation (TMS) treatment in the past 3 months. - Any cognitive remediation therapy in the past 2 years. - Women who are pregnant or breast feeding. - Intention to become pregnant during the course of the study. - Female participants who are surgically sterilised/hysterectomised or post-menopausal for longer than 2 years are not considered as being of child bearing potential. - Previous enrolment into the current study. |

*SI 3: TMS Machinery*

Real and sham rTMS stimulations were delivered using either the MagPro x100 or the MagPro R30 with theta burst option. Both TMS machinery are manufactured by Tonica Electronik A/S Denmark and distributed by Magventure A/S Denmark. Each machine was used in the same building of the department (MagPro x 100 at Bollingerstrasse 111 and MagPro R30 in Mürtnestrasse 21) for the duration of the trial. Patients were treated at the rTMS machine closest to their patient care.

For real rTMS we used the MCF-B70 coil with transducer head dimensions of 180x116x50/67 and a magnetic field of 12kT/s in coil center at 20mm distance from coil surface and penetration depth (70V/m) of 34.0mm. For sham rTMS we used the MCF-P-B65 coil with transducer head dimensions of 172x94x53. The coil used for the sham rTMS looks and sounds identical to the real rTMS coil but with no magnetic emmissions.

*SI 4: SCRT Material*

Both real and sham SCRT included a total of 16-sessions carried out bi-weekly for 8-weeks with each session lasting 90 minutes. The real SCRT^2^ is a tailored broad-based treatment centered on the Integrative Neurocognitive Therapy manual based on the MATRICS focusing on social cognition and neurocognitive processes. It included 5 modules: 1. Emotion perception and expression; 2: Verbal/Visual and learning memory; 3. Social Perception and Theory of Mind; 4. Working memory; and 5. Social schema. Each module contained an Introductory, Training and Transfer session^1^ (Supplementary Table 2a). Only the MATRICS dimensions most relevant to gesture production could be considered, and the structured procedure such as education, individual compensation strategies (completed using computer-based programs such as CogPack and Marker Software), and transfer to daily life had to be shortened due to the limited intervention time.

In contrast, the sham SCRT did not contain any add-on social cognitive training but rather focused on informing patients of the importance of mindfulness, dietary and sleep hygiene, stress-free environment, and the benefits of the arts and included different activities such as breathing exercises, walking and visiting museums (Supplementary Table 2b).

For both real and sham SCRT the last session (#16) involved reflection of the important components covered during therapy and the patients were encouraged to apply the strategies’ they learned in their daily lives. This will help them to step out of their comfort zone and break any habitual behavioral patterns.

The material and order of the SCRTs were the same across all groups. The sessions were led by a head therapist (V.C.) and a co-therapist and supervised by an INT-expert (D.M.) who trained both therapists. After completion of the SCRT, satisfaction ratings were collected from each patient concerning their experience.

| Supplementary Table 2a: REAL SCRT MODULES | | | | |
| --- | --- | --- | --- | --- |
| **Module** | **Session Type** | **Session** | **Details** | **Objectives** |
|  |  |  |  |  |
| **1. Emotion Perception and Expression** | Introductory | Session 1 | Introduction to emotions and perception. Overview of the Filter Model of Perception. Exercises on emotion recognition. | Understand emotions, learn the Filter Model, and practice recognizing emotions. |
|  | Training | Session 2 | Explore basic emotions and their functions. Group activities on defining and recognizing basic emotions. | Identify basic emotions and recognize them through facial expressions. |
|  | Transfer | Session 3 | Advanced perception through gestures and body language. Exercises on emotional mimicry and sequence analysis. | Improve ability to recognize and interpret emotions via non-verbal cues. |
| **2. Verbal/Visual Learning and Memory** | Introductory | Session 4 | Introduction to visual memory. Focus on visual memory with exercises and introduction of the Memory Model. | Understand visual working memory, manage sensory overload, and improve cognitive function. |
|  | Training | Session 5 | Warm-up exercise for visual memory recall. Techniques for remembering numbers and visual memory aids. | Apply memory techniques, manage distractions, and enhance cognitive flexibility. |
|  | Transfer | Session 6 | Introduction to prospective memory. Strategies for visual memory and object categorization. | Enhance working memory and stress management through practical exercises. |
| **3. Social Perception and Theory of Mind** | Introductory | Session 7 | Overview of social cognitive functions, including social perception and Theory of Mind (ToM). | Understand social cognitive functions, apply ToM, and reflect on social interactions. |
|  | Training | Session 8 | Reflect on personal experiences in social situations. Deepen understanding of ToM and practical group exercises. | Apply perspective-taking strategies and understand personal experiences in social contexts. |
|  | Transfer | Session 9 | Enhance skills in perspective-taking. Apply techniques to understand others' perspectives. | Improve perspective-taking skills and adapt strategies to real-life situations. |
| **4. Working Memory** | Introductory | Session 10 | Develop understanding and skills related to visual working memory. Address sensory overload and selective attention. | Develop visual working memory skills, manage distractions, and apply techniques in daily life. |
|  | Training | Session 11 | Develop and apply strategies to improve working memory. Focus on managing sensory overload and emotional stress. | Apply strategies for improving working memory, manage stress, and evaluate coping strategies. |
|  | Transfer | Session 12 | Exercises to enhance cognitive flexibility and task-switching. Review and apply coping strategies. | Enhance cognitive flexibility and task-switching abilities through practical exercises. |
| **5. Social Schema** | Introductory | Session 13 | Focus on social schemas guiding social behavior. Examples and exercises to highlight automatic thinking and behavior patterns. | Increase awareness of automatic social behaviors and adapt strategies to handle disruptions. |
|  | Training | Session 14 | Identify social rules, roles, and prejudices. Discuss and practice managing stigmatization and behavioral scripts. | Identify and adjust social rules and roles, manage prejudices, and handle stigmatization. |
|  | Transfer | Session 15 | Analyze sequences of images and film clips. Evaluate social skills and understanding of social rules. | Analyze and understand social schemas through image and film exercises, and evaluate social skills. |

| Supplementary Table *2b Sham SCRT Sessions* | | | |
| --- | --- | --- | --- |
| Session | Theme | Purpose | Activities |
|  |  |  |  |
| 1 | Getting to Know Each Other | Establish group rapport | Icebreaker, discussion of group principles, 'group characteristics' exercise, 1-minute introductions. |
| 2 | Relaxation & Meditation | Teaching relaxation techniques | Introduction to Progressive Muscle Relaxation (PMR), discussion on stress and tension, PMR exercise. |
| 3 | Mindful Walking in Nature | Therapeutic effects of nature and mindfulness | Mindful walking in the forest, reflection on nature's impact on health, discussion on nature and well-being. |
| 4 | Movement | Relationship between physical activity and mental health | Discussion of personal movement routines, encouragement of light physical activities, reflection on movement's benefits. |
| 5 | Sleep Hygiene | Improving sleep quality | Teaching 10 rules of sleep hygiene, discussion on sleep habits, reflection on sleep's benefits. |
| 6 | Visit to the Museum of Communication | Communication in the digital age | Museum visit, guided exploration, reflection on media's impact on life and communication. |
| 7 | Media Literacy | Impact of social media and media consumption | Discussion of media experiences, exploration of media benefits and risks. |
| 8 | Mindful Eating and Nutrition | Conscious eating for mental well-being | Teaching healthy eating habits, discussion of mindful consumption, impact on stress and health. |
| 9 | Botanical Garden Visit & Body Scan Meditation | Nature experience and mindfulness | Group walk in botanical garden, body scan meditation for relaxation and self-awareness. |
| 10 | Introduction to Mindfulness | Mindfulness techniques for emotional regulation | Discussion on mindfulness, teaching non-judgmental awareness, application in stress management. |
| 11 | The Power of Music | Music as a therapeutic tool | Discussion on emotional connection to music, rhythm-based activities, reflective listening exercises. |
| 12 | Visit to the Paul Klee Art Center | Creativity and art as therapeutic mediums | Visit to art workshop, creative expression, reflection on art as a tool for emotional release. |
| 13 | Walk by the Aare River | Environmental awareness and mental well-being | Guided nature walk, discussion on conservation, impact of environmental changes on health. |
| 14 | Courage and Role Models | Personal courage and role models | Discussion on courage, reflection on personal experiences and role models. |
| 15 | Visit to the Natural History Museum | Reflection on nature and the environment | Museum visit on animal history and evolution, discussion on human impact on the environment. |

*SI 5: Primary Outcome*

Gesture performance accuracy was measured using the Test of Upper-Limb Apraxia (TULIA)^3^. The TULIA test was developed in accordance with the key domains and semantic features of gesture performance. The TULIA measures performance accuracy of hand and finger movements in 48-items across two domains and three semantic categories of gestures. The two domains include Imitation, which involves gesture performance following visual demonstration from the experimenter, and Pantomime, which involves gesture performance following verbal command from the experimenter. Each domain consists of three semantic categories of gestures: Meaningless, which are novel gestures without any semantic features; Intransitive, which are highly learned communicative gestures; and Transitive, which are tool-based gestures.

The TULIA test requires the patient and the experimenter to sit vis-à-vis with a table between them, both with their hands flat on the table. For the imitation domain patients are instructed to perform the required gesture only after the experimenter finished with the demonstration in a mirrored manner and return their performing hand to the original position. For the pantomime domain, the experimenter verbally instructs the patients to perform the required gestures and waits for the patients to return their performing hand to the original position before proceeding to the next gesture item. Patients performed the gestures with their right hand, and were informed on the nature of the gestures following each category. The starting domain and category were randomized across all patients and timepoints.

The TULIA test was videorecorded and later quantified by an independent experimenter who was blinded to the treatment arms and was trained by the principal investigator (S.W.) in accordance to the TULIA manual from Vanbelligen et al^3^. The score for each gesture item was rated using a 0-5 Likert scale. Content errors such body-part-as-objects and perseverations scored 0-2, temporal and spatial errors scored 3-4, while precise performance scored 5. The maximum total score for TULIA is 240 (120 per domain; 40 per category) denoting superior gesture performance.

*SI 6: Secondary outcomes and subscores*

We used the Mini-Profile of Nonverbal Sensitivity (Mini-PONS^4^) to assess nonverbal social perception. The task includes 64 videos of a Caucasian female actor performing different nonverbal cues, and patients’ task was to evaluate these cues in order to pick the best scenario that describes the scene in the video from two options provided. The Mini-PONS includes the performance of nonverbal cues across 4 different subdomains (emotional prosody together with facial expression, emotional prosody alone, facial expressions alone and bodily movements). The test was administered on a computer. The maximum Mini-PONS total score ranges from 0-64 (0-16 per subdomain), with higher values indicative of superior social cognition.

We used the Postural Knowledge Test^5, 6^ to test patients’ general knowledge on the structure of different hand postures. The test consists of 20 cartoon images of people carrying out different gestures. The distal parts of the hand performing these gestures are missing, and patients’ task is to choose the correct hand position based on three options provided below each cartoon image. The test was administered on a computer. The PKT score ranges from 0-20 with higher scores indicative of superior gestural knowledge.

To assess patients’ ability to regulate their own and others’ emotions we used the Mayer-Salovey-Caruso Emotional Intelligence Test (MSCEIT) Managing Emotion branch^7^; one of the four main branches that make-up the MSCEIT. The Managing Emotion branch is divided into two components: the emotion management, which involves the ability to regulate one’s own emotions, and the social management, which focuses on how well one can manage and influence the emotional states of others^7^. A higher score is indicative of better emotional regulation.

Social functioning was measured with standardized rating scales such as the SOFAS and PSP. The SOFAS for example, provides a single global score ranging from 0 to 100 to reflect an individual's overall level of social and occupational functioning, independent of symptom severity^8^. The PSP on the other hand, is a new version of the SOFAS developed to address socially useful activities, personal and social relationships, self-care, and disturbing or aggressive behaviours. It also uses a 100-point scale, interpreted in 10-point intervals, allowing for greater granularity in tracking changes over time compared to SOFAS^8^.

*SI 7. Randomization and allocation concealment*

Considering that the same head therapist (V.C.) administered the therapy to both real and SCRT groups, both groups could not be treated at the same time. Rather, we divided the SCRT administration into separate time blocks.

Prior to enrollment of the first patient, we used ‘research randomizer’ a web-based tool to perform permutated block randomization. Lists with random order of the real SCRT treatment arms were generated, ensuring that both treatment arms received the same number of patients. We did this after recruiting 6-8 patients. In a different time-block, we assigned another 6-8 patients to the sham SCRT. Only the principal investigator (S.W.) and postdoctoral supervisor (A.P.) had access to the randomization protocol.

Group allocation took place after patients provided informed consent, and before the start of any baseline assessments, to ensure no information on outcomes was available before group allocation.

The principal investigator and postdoctoral supervisor were not involved in the screening or recruitment of patients, but rather informed the team member responsible in conducting rTMS and SCRT (V.C.) about group allocation. The psychiatrist (L.M.) involved in conducting the clinical assessments had no access to the randomization lists and was blinded to the treatment arms at all times throughout the trial. In case of emergency the principal investigator could have informed the clinical psychiatrist of the treatment allocation and the study would have stopped in this participant. During the course of the trial two serious adverse events (SAE) were reported. Once informed the principal investigator in turn informed the psychiatrist of the study who evaluated both patients. To determine whether the SAEs were caused by our treatments the psychiatrist was unblinded only for these two patients. Both SAEs were deemed to be unrelated to our treatment arms and the Ethics committee of the canton of Bern, Switzerland was informed immediately. One patient returned for Week-8 and Week-32 assessments, while the other withdrew their consent. Please also see chapter 6 of the study protocol for additional information regarding randomization, blinding and unblinding procedures.

*SI 8. Binomial logistic regression*

To assess the likelihood of the occurrence of any adverse events for our rTMS treatment arms across rTMS sessions we employed a binomial logistic regression instead of a repeated measures ANOVA as stated in the study protocol (Supplement 1). We consider this model more appropriate since our dependent variable is binary and a binomial logistic regression is designed specifically to model binary outcomes by estimating the probability of one outcome, in our case an adverse event, relative to the other^9^. Fixed effects included treatment arms (real rTMS and real SCRT and sham rTMS and real SCRT), rTMS sessions (Session 1-10) and their interaction, with a radom intercept for patients to account for repeated measures. We used the first rTMS session as the reference category allowing for comparisons of all other rTMS sessions (Supplementary Table 7).

*SI 9. Adverse events after SCRT treatment*

Immediately after completion of the last SCRT session we asked all patients to rate their experience and behaviors within their group setting (real or sham SCRT) using a self-reported questionnaire “Experience and Behavior in (Therapy) Groups*”* (EBIT^10^). Importantly, all three conditions involved group-based interventions, ensuring that group effects were present across all comparison groups. The EBIT includes 13 statements, 8 of which are positive such as “I feel comfortable” and “I talk with others in the group”, and 5 of which are negative such as “I feel afraid” and “I am unable to speak”. Patients were asked to rate each statement using a 1-6 Likert scale: 1- Strongly Agree, 2- Agree, 3- Somewhat Agree, 4- Somewhat disagree, 5- Disagree, 6- Strongly disagree. To assess adverse effects of SCRT treatment we focused only on the negative statements. We reversed the score, and compared patients’ responses separately for each statement to assess comfort levels, engagement, anxiety and communication tendencies between treatment arms (Supplementary Table 8).

*Supplementary Results*

*Supplementary Table 3: Percentage of attendance per treatment arm*

|  | **N (%)** |  |  |
| --- | --- | --- | --- |
| **Attendance** | **real rTMS + real SCRT (n=19)** | **sham rTMS + real SCRT (n=26)** | **Sham SCRT (n=28)** |
| **rTMS (10 sessions)** |  |  |  |
| **Completed all** | 15 (79.1%) | 15 (57.7%) | / |
| **=> 75 %** | 3 (15.7%) | 5 (19.2%) | / |
| **=> 50 %** | / | 2 (7.7%) | / |
| **=> 25 %** | / | 2 (7.7%) | / |
| **=> 5 %** | 1 (5.2%) | 2 (7.7%) | / |
| **0** | / | / | / |
| **SCRT (16 sessions)** |  |  |  |
| **Completed all** | 4 (21.1%) | 3 (11.5%) | 7 (25%) |
| **=> 75 %** | 11 (57.9%) | 9 (34.6%) | 14 (50%) |
| **=> 50 %** | 2 (10.5%) | 6 (23.1%) | / |
| **=> 25 %** | 1 (5.2%) | 4 (15.4%) | 2 (7.1%) |
| **=> 5 %** | 1 (5.2%) | 3 (11.5%) | 5 (17.9%) |
| **0** | / | 1 (3.8%) | / |
| Abbreviations: rTMS, repeated Transcranial Magnetic Stimulation; SCRT, Social Cognitive Remediation Therapy | | | |

*Supplementary Table 4: Dropout Reasons for ITT*

| Reason | Real rTMS  + real SCRT | Sham rTMS  + real SCRT | Sham SCRT | Total |
| --- | --- | --- | --- | --- |
| Withdrew consent | 1 | 5 | 6 | 12 |
| Lost to follow-up | 2 | 6 | 0 | 8 |
| Adverse Event | 0 | 0 | 0 | 0 |
| Abbreviations: rTMS, repeated Transcranial Magnetic Stimulation; SCRT, Social Cognitive Remediation Therapy | | | | |

*Supplementary Table 5: Primary and secondary outcomes according to treatment arm for ITT with LOCF until Week-8*

|  | Mean ± SEM | | |  |  |
| --- | --- | --- | --- | --- | --- |
| **Outcomes** | **Real rTMS**  **+ real SCRT** | **Sham rTMS**  **+ real SCRT** | **Sham SCRT** | **ANOVA** | **Posthoc** |
| **TULIA Total** |  |  |  |  | Timepoint: |
|  |  |  |  | Timepoint: | Baseline-Week-2 |
| **Baseline** | 192.7 ± 3.9 | 191.1 ± 4.4 | 193.0 ± 3.0 | F_(2, 140)_ = 5.0, p = 0.001* | t = -1.1, p = 0.3 |
| **Week-2** | 193.4 ± 4.1 | 189.4 ± 4.4 | 197.0 ± 2.6 | Timepoint x arm: | Baseline-Week-8 |
| **Week-8** | 196.3 ± 3.6 | 193.5 ± 4.3 | 197.1 ± 2.9 | F_(4, 140)_ = 1.4, p = 0.3 | t = -3.1, p = 0.01* |
|  |  |  |  |  | Week-2-Week-8 |
|  |  |  |  |  | t = -2.1, p = 0.07 |
| **Imitation** |  |  |  | Timepoint |  |
| **Baseline** | 101.6 ± 1.7 | 100.1 ± 2.1 | 101.7 ± 1.3 | F_(2, 140)_ = 0.8, p = 0.4 |  |
| **Week-2** | 100.2 ± 1.9 | 98.9 ± 1.9 | 102.0 ± 1.5 | Timepoint x arm: |  |
| **Week-8** | 100.8 ± 1.8 | 100.8 ± 1.9 | 101.9 ± 1.3 | F_(4, 140)_ = 0.6, p = 0.7 |  |
| **Pantomime** |  |  |  |  | Timepoint: |
|  |  |  |  | Timepoint: | Baseline-Week-2 |
| **Baseline** | 91.1 ± 2.6 | 91.0 ± 2.8 | 91.2 ± 2.0 | F_(2, 140)_ = 8.3, p = 0.0003* | t = -2.3, p = 0.04* |
| **Week-2** | 93.2 ± 2.7 | 90.6 ± 3.0 | 95.0 ± 1.7 | Timepoint x arm: | Baseline-Week-8 |
| **Week-8** | 95.4 ± 2.4 | 92.7 ± 2.8 | 95.1 ± 1.9 | F_(4, 140)_ = 1.5, p = 0.2 | t = -4.0, p = 0.0003* |
|  |  |  |  |  | Week-2-Week-8 |
|  |  |  |  |  | t = -1.7, p = 0.08 |
| **Imitation_meaningless_** |  |  |  | Timepoint |  |
| **Baseline** | 35.2 ± 0.6 | 34.6 ± 0.7 | 35.5 ± 0.6 | F_(2, 140)_ = 0.3, p = 0.8 |  |
| **Week-2** | 34.7 ± 0.7 | 35.3 ± 0.5 | 35.0 ± 0.6 | Timepoint x arm: |  |
| **Week-8** | 34.3 ± 0.7 | 34.7 ± 0.7 | 35.4 ± 0.5 | F_(4, 140)_ = 1.0, p = 0.4 |  |
| **Imitation_Intransitive_** |  |  |  | Timepoint |  |
| **Baseline** | 35.4 ± 0.6 | 34.4 ± 0.8 | 34.8 ± 0.5 | F_(2, 140)_ = 1.8, p = 0.2 |  |
| **Week-2** | 34.2 ± 0.7 | 33.4 ± 0.6 | 35.1 ± 0.6 | Timepoint x arm: |  |
| **Week-8** | 35.2 ± 0.7 | 34.8 ± 0.7 | 34.5 ± 0.6 | F_(4, 140)_ = 1.9, p = 0.1 |  |
| **Imitation_transitive_** |  |  |  | Timepoint |  |
| **Baseline** | 31.0 ± 1.1 | 31.1 ± 0.9 | 31.4 ± 0.6 | F_(2, 140)_ = 1.0, p = 0.4 |  |
| **Week-2** | 31.2 ± 0.9 | 30.1 ± 1.0 | 31.8 ± 0.7 | Timepoint x arm: |  |
| **Week-8** | 31.3 ± 0.8 | 31.3 ± 0.9 | 32.0 ± 0.7 | F_(4, 140)_ = 0.8, p = 0.5 |  |
| **Pantomime_meaningless_** |  |  |  |  | Timepoint: |
|  |  |  |  | Timepoint | Baseline-Week-2 |
| **Baseline** | 30.9 ± 1.2 | 31.0 ± 1.4 | 32.1± 1.0 | F_(2, 140)_ = 4.3, p = 0.02* | t = -2.5, p = 0.02* |
| **Week-2** | 33.3 ± 1.0 | 31.2 ± 1.4 | 33.2 ± 0.8 | Timepoint x arm: | Baseline-Week-8 |
| **Week-8** | 33.5 ± 0.9 | 31.9 ± 1.3 | 32.5 ± 0.9 | F_(4, 140)_ = 1.5, p = 0.2 | t = -2.6, p = 0.02* |
|  |  |  |  |  | Week-2-Week-8 |
|  |  |  |  |  | t = -0.6, p = 0.9 |
| **Pantomime_Intransitive_** |  |  |  |  | Timepoint: |
|  |  |  |  | Timepoint | Baseline-Week-2 |
| **Baseline** | 31.1 ± 0.9 | 30.1 ± 0.9 | 30.5 ± 0.7 | F_(2, 140)_ = 8.2, p = 0.0004* | t = -1.0, p = 0.3 |
| **Week-2** | 31.0 ± 1.3 | 30.2 ± 1.0 | 31.5 ± 0.8 | Timepoint x arm: | Baseline-Week-8 |
| **Week-8** | 32.5 ± 1.0 | 30.7 ± 1.1 | 32.8 ± 0.8 | F_(4, 140)_ = 1.1, p = 0.3 | t = -3.9, p = 0.0004* |
|  |  |  |  |  | Week-2-Week-8 |
|  |  |  |  |  | t = -2.9, p = 0.007* |
| **Pantomime_transitive_** |  |  |  | Timepoint |  |
| **Baseline** | 29.1 ± 1.3 | 29.9 ± 0.9 | 28.5 ± 0.9 | F_(2, 140)_ = 0.9, p = 0.4 |  |
| **Week-2** | 28.9 ± 1.1 | 29.1 ± 1.0 | 30.2 ± 1.0 | Timepoint x arm: |  |
| **Week-8** | 29.3 ± 1.1 | 30.1 ± 0.7 | 29.7 ± 1.0 | F_(4, 140)_ = 1.7, p = 0.2 |  |
| **Mini-PONS total^a^** |  |  |  | Timepoint |  |
| **Baseline** | 42.4 ± 1.3 | 42.0 ± 1.1 | 43.5 ± 1.5 | F_(2, 138)_ = 0.8, p = 0.5 |  |
| **Week-2** | 42.1 ± 1.1 | 43.5 ± 1.0 | 43.9 ± 1.1 | Timepoint x arm: |  |
| **Week-8** | 41.8 ± 1.0 | 43.1 ± 1.0 | 43.4 ± 1.4 | F_(4, 138)_ = 0.6, p = 0.6 |  |
| **Mini-PONS_face_^a^** |  |  |  | Timepoint |  |
| **Baseline** | 10.8 ± 0.4 | 11.1 ± 0.4 | 10.8 ± 0.4 | F_(2, 138)_ = 2.0, p = 0.1 |  |
| **Week-2** | 11.1 ± 0.4 | 11.5 ± 0.3 | 11.3 ± 0.3 | Timepoint x arm: |  |
| **Week-8** | 10.8 ± 0.4 | 11.3 ± 0.4 | 11.4 ± 0.4 | F_(4, 138)_ = 0.4, p = 0.8 |  |
| **Mini-PONS_hands_^a^** |  |  |  | Timepoint |  |
| **Baseline** | 10.6 ± 0.5 | 9.8 ± 0.4 | 11.0 ± 0.5 | F_(2, 138)_ = 0.04, p = 0.9 |  |
| **Week-2** | 10.4 ± 0.4 | 10.0 ± 0.4 | 10.6 ± 0.4 | Timepoint x arm: |  |
| **Week-8** | 10.4 ± 0.4 | 10.1 ± 0.5 | 10.9 ± 0.5 | F_(4, 138)_ = 0.3, p = 0.8 |  |
| **Mini-PONS_voice_^a^** |  |  |  | Timepoint |  |
| **Baseline** | 10.5 ± 0.5 | 9.8 ± 0.4 | 10.3 ± 0.4 | F_(2, 138)_ = 0.3, p = 0.7 |  |
| **Week-2** | 9.7 ± 0.4 | 10.3 ± 0.4 | 10.3 ± 0.4 | Timepoint x arm: |  |
| **Week-8** | 9.3 ± 0.4 | 10.2 ± 0.4 | 10.2 ± 0.4 | F_(4, 138)_ = 2.0, p = 0.1 |  |
| **Mini-PONS_face+voice_^a^** |  |  |  | Timepoint |  |
| **Baseline** | 10.4 ± 0.5 | 11.3 ± 0.4 | 11.5 ± 0.6 | F_(2, 138)_ = 0.9, p = 0.4 |  |
| **Week-2** | 10.8 ± 0.5 | 11.7 ± 0.4 | 11.3 ± 0.4 | Timepoint x arm: |  |
| **Week-8** | 11.2 ± 0.4 | 11.5 ± 0.3 | 11.0 ± 0.5 | F_(4, 138)_ = 1.2, p = 0.3 |  |
| **PKT Total** |  |  |  | Timepoint |  |
| **Baseline** | 14.4 ± 0.9 | 14.5 ± 0.9 | 14.5 ± 0.6 | F_(2, 140)_ = 1.8, p = 0.4 |  |
| **Week-2** | 14.7 ± 1.0 | 14.5 ± 0.8 | 15.5 ± 0.4 | Timepoint x arm: |  |
| **Week-8** | 15.1 ± 0.7 | 14.7 ± 0.8 | 15.0 ± 0.5 | F_(4, 140)_ = 0.7, p = 0.6 |  |
| **MSCEIT_Managing Emotion_^b^** |  |  |  | Timepoint |  |
| **Baseline** | 86.0 ± 2.1 | 88.8 ± 1.9 | 84.4 ± 1.8 | F_(1, 68)_ = 0.1, p = 0.7 |  |
| **Week 8** | 84.2± 2.3 | 87.7 ± 2.1 | 85.7 ± 2.0 | Timepoint x arm: |  |
|  |  |  |  | F_(2, 68)_ = 1.2, p = 0.3 |  |
| **MSCEIT_Emotion Management_^b^** |  |  |  | Timepoint |  |
| **Baseline** | 87.4 ± 2.0 | 89.5 ± 1.7 | 86.7 ± 1.7 | F_(1, 68)_ = 1.1, p = 0.3 |  |
| **Week 8** | 87.8 ± 2.4 | 90.0 ± 2.0 | 88.9 ± 2.0 | Timepoint x arm: |  |
|  |  |  |  | F_(2, 68))_ = 0.3, p = 0.7 |  |
| **MSCEIT_Social Management_^b^** |  |  |  | Timepoint |  |
| **Baseline** | 87.1 ± 2.1 | 89.5 ± 1.9 | 85.0 ± 1.8 | F_(1, 68))_ = 2.7, p = 0.1 |  |
| **Week 8** | 84.3± 1.9 | 87.6 ± 1.8 | 85.2 ± 1.8 | Timepoint x arm: |  |
|  |  |  |  | F_(2, 68))_ = 1.3, p = 0.3 |  |
| **PANSS Positive** |  |  |  |  | Timepoint: |
|  |  |  |  | Timepoint | Baseline-Week-2 |
| **Baseline** | 16.2 ± 1.7 | 12.6 ± 1.0 | 13.9 ± 0.8 | F_(2, 140)_ = 5.6, p = 0.005* | t = 1.7, p = 0.1 |
| **Week-2** | 15.0 ± 1.5 | 11.6 ± 0.7 | 13.6 ± 0.9 | Timepoint x arm | Baseline-Week-8 |
| **Week-8** | 14.3 ± 1.3 | 11.8 ± 0.7 | 12.4 ± 0.8 | F_(4, 140)_ = 0.4, p = 0.8 | t = 3.3, p = 0.003* |
|  |  |  |  |  | Week-2-Week-8 |
|  |  |  |  |  | t = 1.6, p = 0.1 |
| **PANSS Negative** |  |  |  |  | Timepoint: |
|  |  |  |  | Timepoint | Baseline-Week-2 |
| **Baseline** | 20.1 ± 1.9 | 17.7 ± 1.7 | 15.3 ± 1.2 | F_(2, 140)_ = 3.2, p = 0.04* | t = 2.0, p = 0.07 |
| **Week-2** | 17.9 ± 1.7 | 15.5 ± 1.5 | 15.2 ± 1.1 | Timepoint x arm | Baseline-Week-8 |
| **Week-8** | 16.5 ± 1.7 | 16.3 ± 1.5 | 14.8 ± 1.5 | F_(4, 140)_ = 0.6, p = 0.7 | t = 2.3, p = 0.06 |
|  |  |  |  |  | Week-2-Week-8 |
|  |  |  |  |  | t = 0.3, p = 0.7 |
| **PANSS Total** |  |  |  |  | Timepoint: |
|  |  |  |  | Timepoint | Baseline-Week-2 |
| **Baseline** | 72.2 ± 6.0 | 60.9 ± 3.9 | 59.9 ± 2.7 | F_(2, 140)_ = 6.5, p = 0.002* | t = 2.9, p = 0.005* |
| **Week-2** | 62.2 ± 4.5 | 54.6 ± 3.6 | 58.9 ± 2.7 | Timepoint x arm | Baseline-Week-8 |
| **Week-8** | 60.5 ± 5.0 | 56.5 ± 3.4 | 57.2 ± 3.3 | F_(4, 140)_ = 1.6, p = 0.2 | t = 3.2, p = 0.004* |
|  |  |  |  |  | Week-2-Week-8 |
|  |  |  |  |  | t = 0.2, p = 0.8 |
| **BNSS Total** |  |  |  |  | Timepoint: |
|  |  |  |  | Timepoint | Baseline-Week-2 |
| **Baseline** | 33.9 ± 3.9 | 27.8 ± 3.2 | 26.7 ± 3.2 | F_(2, 140)_ = 3.1, p = 0.04* | t = 1.5, p = 0.2 |
| **Week-2** | 30.7 ± 4.0 | 24.3 ± 3.2 | 26.0 ± 3.0 | Timepoint x arm: | Baseline-Week-8 |
| **Week-8** | 28.4 ± 4.3 | 24.8 ± 3.3 | 23.5 ± 2.8 | F_(4, 140)_ = 0.4, p = 0.8 | t = 2.5, p = 0.04* |
|  |  |  |  |  | Week-2-Week-8 |
|  |  |  |  |  | t = 0.9, p = 0.4 |
| **SNS Total^c^** |  |  |  | Timepoint |  |
| **Baseline** | 14.6 ± 1.5 | 15.8 ± 1.8 | 16.7 ± 1.2 | F_(2, 132)_ = 1.5, p = 0.2 |  |
| **Week-2** | 15.7 ± 1.8 | 14.4 ± 1.8 | 16.7 ± 1.1 | Timepoint x arm: |  |
| **Week-8** | 13.5 ± 1.7 | 13.9 ± 2.0 | 16.8 ± 1.5 | F_(4, 132)_ = 1.3, p = 0.3 |  |
| **BAG mean Total^d^** |  |  |  | Timepoint |  |
| **Baseline** | 3.1 ± 0.1 | 3.1 ± 0.1 | 3.1 ± 0.1 | F_(2, 128)_ = 1.6, p = 0.2 |  |
| **Week-2** | 3.3 ± 0.1 | 3.2 ± 0.1 | 3.1 ± 0.1 | Timepoint x arm: |  |
| **Week-8** | 3.1 ± 0.1 | 3.2 ± 0.1 | 3.1 ± 0.1 | F_(4, 128)_ = 1.0, p = 0.4 |  |
| **SOFAS Total** |  |  |  |  | Timepoint: |
|  |  |  |  | Timepoint | Baseline-Week-8 |
| **Baseline** | 44.4 ± 2.4 | 51.6 ± 2.5 | 47.9 ± 1.8 | F_(1, 70)_ = 15.4, p = 0.0002* | t = -3.7, p = 0.0005* |
| **Week-8** | 57.7 ± 3.8 | 54.2 ± 2.6 | 50.3 ± 2.1 | Timepoint x arm: | Timepoint x arm: |
|  |  |  |  | F_(2, 70)_ = 6.3, p = 0.003* | Real rTMS real SCRT |
|  |  |  |  |  | Baseline-Week-8 |
|  |  |  |  |  | t = -5.1, p = 0.0001* |
|  |  |  |  |  | sham rTMS real SCRT |
|  |  |  |  |  | Baseline-Week-8 |
|  |  |  |  |  | t = -1.1, p = 0.3 |
|  |  |  |  |  | Sham SCRT |
|  |  |  |  |  | Baseline-Week-8 |
|  |  |  |  |  | t = -1.1, p = 0.3 |
| **SLOF Total** |  |  |  |  |  |
|  |  |  |  | Timepoint | Timepoint: |
| **Baseline** | 174.9 ± 5.8 | 183.7 ± 3.5 | 179.1 ± 3.9 | F_(1, 70)_ = 9.3, p = 0.003* | Baseline-Week-8 |
| **Week-8** | 184.8 ± 5.3 | 190.0 ± 3.1 | 183.0 ± 3.2 | Timepoint x arm: | t = -3.1, p = 0.003* |
|  |  |  |  | F_(2, 70)_ = 0.6, p = 0.5 |  |
|  |  |  |  |  |  |
| **PSP Total** |  |  |  |  | Timepoint: |
|  |  |  |  | Timepoint | Baseline-Week-8 |
| **Baseline** | 46.1 ± 2.7 | 51.4 ± 2.5 | 47.1 ± 2.0 | F_(1, 70)_ = 11.0, p = 0.001* | t = -3.2, p = 0.002* |
| **Week-8** | 57.2 ± 3.7 | 54.2 ± 2.7 | 49.4 ± 2.5 | Timepoint x arm: | Timepoint x arm: |
|  |  |  |  | F_(2, 70)_ = 3.5, p = 0.03* | Real rTMS real SCRT |
|  |  |  |  |  | Baseline-Week-8 |
|  |  |  |  |  | t = -3.9, p = 0.003* |
|  |  |  |  |  | sham rTMS real SCRT |
|  |  |  |  |  | Baseline-Week-8 |
|  |  |  |  |  | t = -1.1, p = 0.4 |
|  |  |  |  |  | Sham SCRT |
|  |  |  |  |  | Baseline-Week-8 |
|  |  |  |  |  | t = -0.9, p = 0.4 |
| **UPSA-B Total** |  |  |  | Timepoint |  |
| **Baseline** | 74.4 ± 6.0 | 76.3 ± 4.2 | 79.6 ± 2.2 | F_(1, 70)_ = 0.8, p = 0.4 |  |
| **Week-8** | 80.5 ± 4.1 | 76.2 ± 4.3 | 78.7 ± 2.8 | Timepoint x arm: |  |
|  |  |  |  | F_(2, 70)_ = 2.2, p = 0.1 |  |
| \| \| Note: rTMS: Repetitive Trnascranial Magnetic Stimulation; SCRT: Social Cognitive Remediation Therapy. ^a^one patient from real rTMS and real SCRT missing Mini-PONS; ^b^two patients from sham rTMS and real SCRT missing MSCEIT; ^c^one patient from real rTMS and real SCRT, one patient from sham rTMS and real SCRT, and two patients from sham SCRT missing SNS; ^d^two patients from real rTMS and real SCRT, two patients from sham rTMS and real SCRT, and two patients from sham SCRT missing BAG; * denotes a significant effect \| \| --- \| \| \| --- \| --- \| | | | | | |

*Supplementary Table 6: Primary and secondary outcomes according to treatment arm for ITT with LOCF until Week-32 follow-up*

|  | Mean ± SEM | | |  |  |
| --- | --- | --- | --- | --- | --- |
| **Outcomes** | **Real rTMS**  **+ real SCRT** | **Sham rTMS**  **+ real SCRT** | **Sham SCRT** | **ANOVA** | **Posthoc** |
| **TULIA Total** |  |  |  |  | Timepoint: |
|  |  |  |  | Timepoint: | Baseline-Week-2 |
| **Baseline** | 192.7 ± 3.9 | 191.1 ± 4.4 | 193.0 ± 3.0 | F_(3, 210)_ = 5.8, p = 0.0008* | t = -1.1, p = 0.3 |
| **Week-2** | 193.4 ± 4.1 | 189.4 ± 4.4 | 197.0 ± 2.6 | Timepoint x arm: | Baseline-Week-8 |
| **Week-8** | 196.3 ± 3.6 | 193.5 ± 4.3 | 197.1 ± 2.9 | F_(6, 210)_ = 1.2, p = 0.3 | t = -3.2, p = 0.01* |
| **Week-32** | 197.3 ± 3.7 | 192.3 ± 4.3 | 198.5 ± 2.7 |  | Baseline-Week-32 |
|  |  |  |  |  | t = -3.6, p = 0.003* |
|  |  |  |  |  | Week-2-Week-8 |
|  |  |  |  |  | t = -2.1, p = 0.06 |
|  |  |  |  |  | Week-2–Week-32 |
|  |  |  |  |  | t = -2.5, p = 0.03* |
|  |  |  |  |  | Week-8-Week-32 |
|  |  |  |  |  | t = 0.4, p = 0.7 |
| **Imitation** |  |  |  | Timepoint |  |
| **Baseline** | 101.6 ± 1.7 | 100.1 ± 2.1 | 101.7 ± 1.3 | F_(3, 210)_ = 1.0, p = 0.4 |  |
| **Week-2** | 100.2 ± 1.9 | 98.9 ± 1.9 | 102.0 ± 1.5 | Timepoint x arm: |  |
| **Week-8** | 100.8 ± 1.8 | 100.8 ± 1.9 | 101.9 ± 1.3 | F_(6, 210)_ = 0.7, p = 0.7 |  |
| **Week-32** | 100.6 ± 1.8 | 100.3 ± 1.9 | 103.4 ± 1.1 |  |  |
| **Pantomime** |  |  |  |  | Timepoint: |
|  |  |  |  | Timepoint: | Baseline-Week-2 |
| **Baseline** | 91.1 ± 2.6 | 91.0 ± 2.8 | 91.2 ± 2.0 | F_(3, 210)_= 8.7, p = 0.00008* | t = -2.5, p = 0.03* |
| **Week-2** | 93.2 ± 2.7 | 90.6 ± 3.0 | 95.0 ± 1.7 | Timepoint x arm: | Baseline-Week-8 |
| **Week-8** | 95.4 ± 2.4 | 92.7 ± 2.8 | 95.1 ± 1.9 | F_(6, 210)_ = 1.8, p = 0.1 | t = -4.3, p = 0.0001* |
| **Week-32** | 96.7 ± 2.6 | 92.0 ± 2.7 | 95.1 ± 2.0 |  | Baseline-Week-32 |
|  |  |  |  |  | t = -4.9, p = 0.0001* |
|  |  |  |  |  | Week-2-Week-8 |
|  |  |  |  |  | t = -1.9, p = 0.08 |
|  |  |  |  |  | Week-2–Week-32 |
|  |  |  |  |  | t = -2.0, p = 0.07 |
|  |  |  |  |  | Week-8-Week-32 |
|  |  |  |  |  | t = 0.1, p = 0.9 |
| **Imitation_meaningless_** |  |  |  | Timepoint |  |
| **Baseline** | 35.2 ± 0.6 | 34.6 ± 0.7 | 35.5 ± 0.6 | F_(3, 210)_ = 0.2, p = 0.9 |  |
| **Week-2** | 34.7 ± 0.7 | 35.3 ± 0.5 | 35.0 ± 0.6 | Timepoint x arm: |  |
| **Week-8** | 34.3 ± 0.7 | 34.7 ± 0.7 | 35.4 ± 0.5 | F_(6, 210)_ = 1.2, p = 0.3 |  |
| **Week-32** | 34.4 ± 0.6 | 34.5 ± 0.7 | 35.9 ± 0.4 |  |  |
| **Imitation_Intransitive_** |  |  |  | Timepoint |  |
| **Baseline** | 35.4 ± 0.6 | 34.4 ± 0.8 | 34.8 ± 0.5 | F_(3, 210)_ = 1.3, p = 0.3 |  |
| **Week-2** | 34.2 ± 0.7 | 33.4 ± 0.6 | 35.1 ± 0.6 | Timepoint x arm: |  |
| **Week-8** | 35.2 ± 0.7 | 34.8 ± 0.7 | 34.5 ± 0.6 | F_(6, 210)_ = 1.4, p = 0.2 |  |
| **Week-32** | 34.8 ± 0.7 | 34.5 ± 0.7 | 34.9 ± 0.6 |  |  |
| **Imitation_transitive_** |  |  |  | Timepoint |  |
| **Baseline** | 31.0 ± 1.1 | 31.1 ± 0.9 | 31.4 ± 0.6 | F_(3, 210)_ = 1.4, p = 0.3 |  |
| **Week-2** | 31.2 ± 0.9 | 30.1 ± 1.0 | 31.8 ± 0.7 | Timepoint x arm: |  |
| **Week-8** | 31.3 ± 0.8 | 31.3 ± 0.9 | 32.0 ± 0.7 | F_(6, 210)_ = 0.7, p = 0.7 |  |
| **Week-32** | 31.3 ± 1.0 | 31.3 ± 0.9 | 32.5 ± 0.7 |  |  |
| **Pantomime_meaningless_** |  |  |  |  | Timepoint: |
|  |  |  |  | Timepoint | Baseline-Week-2 |
| **Baseline** | 30.9 ± 1.2 | 31.0 ± 1.4 | 32.1± 1.0 | F_(3, 210)_ = 4.0, p = 0.008* | t = -2.7, p = 0.01* |
| **Week-2** | 33.3 ± 1.0 | 31.2 ± 1.4 | 33.2 ± 0.8 | Timepoint x arm: | Baseline-Week-8 |
| **Week-8** | 33.5 ± 0.9 | 31.9 ± 1.3 | 32.5 ± 0.9 | F_(6, 210)_ = 2.2, p = 0.04* | t = -2.8, p = 0.01* |
| **Week-32** | 34.1 ± 0.9 | 31.9 ± 1.3 | 32.2 ± 1.0 |  | Baseline-Week-32 |
|  |  |  |  |  | t = -2.9, p = 0.01* |
|  |  |  |  |  | Week-2-Week-8 |
|  |  |  |  |  | t = -0.1, p = 0.9 |
|  |  |  |  |  | Week-2–Week-32 |
|  |  |  |  |  | t = -0.1, p = 0.9 |
|  |  |  |  |  | Week-8-Week-32 |
|  |  |  |  |  | t = -0.1, p = 0.9 |
|  |  |  |  |  | Timepoint x arm: |
|  |  |  |  |  | Real rTMS real SCRT |
|  |  |  |  |  | Baseline-Week-2 |
|  |  |  |  |  | t = -3.0, p = 0.07 |
|  |  |  |  |  | Baseline-Week-8 |
|  |  |  |  |  | t = -3.3, p = 0.04* |
|  |  |  |  |  | Baseline-Week-32 |
|  |  |  |  |  | t = -4.0, p = 0.006* |
|  |  |  |  |  | Week-2-Week-8 |
|  |  |  |  |  | t = -0.3, p = 0.9 |
|  |  |  |  |  | Week-2–Week-32 |
|  |  |  |  |  | t = -1.0, p = 0.8 |
|  |  |  |  |  | Week-8-Week-32 |
|  |  |  |  |  | t = 0.8, p = 0.8 |
|  |  |  |  |  | Sham rTMS real SCRT |
|  |  |  |  |  | Baseline-Week-2 |
|  |  |  |  |  | t = -0.3, p = 0.9 |
|  |  |  |  |  | Baseline-Week-8 |
|  |  |  |  |  | t = -1.4, p = 0.8 |
|  |  |  |  |  | Baseline-Week-32 |
|  |  |  |  |  | t = -1.4, p = 0.8 |
|  |  |  |  |  | Week-2-Week-8 |
|  |  |  |  |  | t = -1.0, p = 0.8 |
|  |  |  |  |  | Week-2–Week-32 |
|  |  |  |  |  | t = -1.0, p = 0.8 |
|  |  |  |  |  | Week-8-Week-32 |
|  |  |  |  |  | t = 0.0, p = 1.0 |
|  |  |  |  |  | Sham SCRT |
|  |  |  |  |  | Baseline-Week-2 |
|  |  |  |  |  | t = -1.6, p = 0.8 |
|  |  |  |  |  | Baseline-Week-8 |
|  |  |  |  |  | t = -0.6, p = 0.8 |
|  |  |  |  |  | Baseline-Week-32 |
|  |  |  |  |  | t = -0.1, p = 0.9 |
|  |  |  |  |  | Week-2-Week-8 |
|  |  |  |  |  | t = 1.1, p = 0.8 |
|  |  |  |  |  | Week-2–Week-32 |
|  |  |  |  |  | t = 1.6, p = 0.8 |
|  |  |  |  |  | Week-8-Week-32 |
|  |  |  |  |  | t = -0.5, p = 0.8 |
| **Pantomime_Intransitive_** |  |  |  |  | Timepoint: |
|  |  |  |  | Timepoint | Baseline-Week-2 |
| **Baseline** | 31.1 ± 0.9 | 30.1 ± 0.9 | 30.5 ± 0.7 | F_(3, 210)_ = 9.7, p = 0.00005* | t = -1.1, p = 0.3 |
| **Week-2** | 31.0 ± 1.3 | 30.2 ± 1.0 | 31.5 ± 0.8 | Timepoint x arm: | Baseline-Week-8 |
| **Week-8** | 32.5 ± 1.0 | 30.7 ± 1.1 | 32.8 ± 0.8 | F_(6, 210)_ = 1.6, p = 0.1 | t = -4.2, p = 0.0001* |
| **Week-32** | 33.1 ± 1.0 | 30.5 ± 1.1 | 32.8 ± 0.7 |  | Baseline-Week-32 |
|  |  |  |  |  | t = -4.3, p = 0.0001* |
|  |  |  |  |  | Week-2-Week-8 |
|  |  |  |  |  | t = -3.1, p = 0.003* |
|  |  |  |  |  | Week-2–Week-32 |
|  |  |  |  |  | t = -3.2, p = 0.003* |
|  |  |  |  |  | Week-8-Week-32 |
|  |  |  |  |  | t = 0.1, p = 0.9 |
| **Pantomime_transitive_** |  |  |  | Timepoint |  |
| **Baseline** | 29.1 ± 1.3 | 29.9 ± 0.9 | 28.5 ± 0.9 | F_(3, 210)_ = 0.9, p = 0.4 |  |
| **Week-2** | 28.9 ± 1.1 | 29.1 ± 1.0 | 30.2 ± 1.0 | Timepoint x arm: |  |
| **Week-8** | 29.3 ± 1.1 | 30.1 ± 0.7 | 29.7 ± 1.0 | F_(6, 210)_ = 1.4, p = 0.2 |  |
| **Week-32** | 29.6 ± 1.1 | 29.6 ± 0.8 | 30.1 ± 1.1 |  |  |
| **Mini-PONS total^a^** |  |  |  | Timepoint |  |
| **Baseline** | 42.4 ± 1.3 | 42.0 ± 1.1 | 43.5 ± 1.5 | F_(3, 207)_ = 0.8, p = 0.5 |  |
| **Week-2** | 42.1 ± 1.1 | 43.5 ± 1.0 | 43.9 ± 1.1 | Timepoint x arm: |  |
| **Week-8** | 41.8 ± 1.0 | 43.1 ± 1.0 | 43.4 ± 1.4 | F_(6, 207)_ = 0.5, p = 0.8 |  |
| **Week-32** | 42.3 ± 1.1 | 42.8 ± 1.2 | 44.4 ± 1.0 |  |  |
| **Mini-PONS_face_^a^** |  |  |  | Timepoint |  |
| **Baseline** | 10.8 ± 0.4 | 11.1 ± 0.4 | 10.8 ± 0.4 | F_(3, 207)_ = 1.7, p = 0.2 |  |
| **Week-2** | 11.1 ± 0.4 | 11.5 ± 0.3 | 11.3 ± 0.3 | Timepoint x arm: |  |
| **Week-8** | 10.8 ± 0.4 | 11.3 ± 0.4 | 11.4 ± 0.4 | F_(6, 207)_ = 0.5, p = 0.8 |  |
| **Week-32** | 11.3 ± 0.3 | 11.2 ± 0.4 | 11.2 ± 0.3 |  |  |
| **Mini-PONS_hands_^a^** |  |  |  | Timepoint |  |
| **Baseline** | 10.6 ± 0.5 | 9.8 ± 0.4 | 11.0 ± 0.5 | F_(3, 207)_ = 0.5, p = 0.7 |  |
| **Week-2** | 10.4 ± 0.4 | 10.0 ± 0.4 | 10.6 ± 0.4 | Timepoint x arm: |  |
| **Week-8** | 10.4 ± 0.4 | 10.1 ± 0.5 | 10.9 ± 0.5 | F_(6, 207)_ = 0.4, p = 0.9 |  |
| **Week-32** | 10.2 ± 0.4 | 10.4 ± 0.4 | 11.2 ± 0.4 |  |  |
| **Mini-PONS_voice_^a^** |  |  |  | Timepoint |  |
| **Baseline** | 10.5 ± 0.5 | 9.8 ± 0.4 | 10.3 ± 0.4 | F_(3, 207)_ = 0.4, p = 0.7 |  |
| **Week-2** | 9.7 ± 0.4 | 10.3 ± 0.4 | 10.3 ± 0.4 | Timepoint x arm: |  |
| **Week-8** | 9.3 ± 0.4 | 10.2 ± 0.4 | 10.2 ± 0.4 | F_(6, 207)_ = 1.8, p = 0.08 |  |
| **Week-32** | 9.7 ± 0.5 | 10.1 ± 0.4 | 10.8 ± 0.4 |  |  |
| **Mini-PONS_face+voice_^a^** |  |  |  | Timepoint |  |
| **Baseline** | 10.4 ± 0.5 | 11.3 ± 0.4 | 11.5 ± 0.6 | F_(3, 207)_ = 0.7, p = 0.5 |  |
| **Week-2** | 10.8 ± 0.5 | 11.7 ± 0.4 | 11.3 ± 0.4 | Timepoint x arm: |  |
| **Week-8** | 11.2 ± 0.4 | 11.5 ± 0.3 | 11.0 ± 0.5 | F_(6, 207)_ = 1.0, p = 0.4 |  |
| **Week-32** | 11.2 ± 0.4 | 11.1 ± 0.4 | 11.2 ± 0.4 |  |  |
| **PKT Total** |  |  |  | Timepoint |  |
| **Baseline** | 14.4 ± 0.9 | 14.5 ± 0.9 | 14.5 ± 0.6 | F_(3, 210)_ = 1.7, p = 0.1 |  |
| **Week-2** | 14.7 ± 1.0 | 14.5 ± 0.8 | 15.5 ± 0.4 | Timepoint x arm: |  |
| **Week-8** | 15.1 ± 0.7 | 14.7 ± 0.8 | 15.0 ± 0.5 | F_(6, 210)_ = 0.8, p = 0.3 |  |
| **Week-32** | 15.4 ± 0.7 | 14.5 ± 0.8 | 15.1 ± 0.5 |  |  |
| **MSCEIT_Managing Emotion_^b^** |  |  |  | Timepoint |  |
| **Baseline** | 86.0 ± 2.1 | 88.8 ± 1.9 | 84.4 ± 1.8 | F_(2, 136)_ = 0.1, p = 0.9 |  |
| **Week-8** | 84.2± 2.3 | 87.7 ± 2.1 | 85.7 ± 2.0 | Timepoint x arm: |  |
| **Week-32** | 85.3 ± 2.3 | 88.5 ± 2.1 | 84.8 ± 1.8 | F_(4, 136)_ = 0.8, p = 0.5 |  |
| **MSCEIT_Emotion Management_^b^** |  |  |  | Timepoint |  |
| **Baseline** | 87.4 ± 2.0 | 89.5 ± 1.7 | 86.7 ± 1.7 | F_(2, 136)_ = 0.8, p = 0.5 |  |
| **Week-8** | 87.8 ± 2.4 | 90.0 ± 2.0 | 88.9 ± 2.0 | Timepoint x arm: |  |
| **Week-32** | 88.6 ± 2.2 | 90.1 ± 2.0 | 87.2 ± 1.8 | F_(4, 136)_ = 0.4, p = 0.8 |  |
| **MSCEIT_Social Management_^b^** |  |  |  | Timepoint |  |
| **Baseline** | 87.1 ± 2.1 | 89.5 ± 1.9 | 85.0 ± 1.8 | F_(2, 136)_ = 1.6, p = 0.2 |  |
| **Week-8** | 84.3± 1.9 | 87.6 ± 1.8 | 85.2 ± 1.8 | Timepoint x arm: |  |
| **Week-32** | 15.4 ± 0.7 | 14.5 ± 0.8 | 15.1 ± 0.5 | F_(4, 136)_ = 0.8, p = 0.5 |  |
| **PANSS Positive** |  |  |  |  | Timepoint: |
|  |  |  |  | Timepoint | Baseline-Week-2 |
| **Baseline** | 16.2 ± 1.7 | 12.6 ± 1.0 | 13.9 ± 0.8 | F_(3, 210)_ = 6.0, p = 0.006* | t = 1.8, p = 0.1 |
| **Week-2** | 15.0 ± 1.5 | 11.6 ± 0.7 | 13.6 ± 0.9 | Timepoint x arm: | Baseline-Week-8 |
| **Week-8** | 14.3 ± 1.3 | 11.8 ± 0.7 | 12.4 ± 0.8 | F_(6, 210)_ = 0.8, p = 0.5 | t = 3.4, p = 0.002* |
| **Week-32** | 13.5 ± 1.5 | 11.7 ± 0.6 | 12.4 ± 0.8 |  | Baseline-Week-32 |
|  |  |  |  |  | t = 3.8, p = 0.001* |
|  |  |  |  |  | Week-2-Week-8 |
|  |  |  |  |  | t = 1.6, p = 0.1 |
|  |  |  |  |  | Week-2–Week-32 |
|  |  |  |  |  | t = 2.0, p = 0.08 |
|  |  |  |  |  | Week-8-Week-32 |
|  |  |  |  |  | t = -0.4, p = 0.7 |
| **PANSS Negative** |  |  |  |  | Timepoint: |
|  |  |  |  | Timepoint | Baseline-Week-2 |
| **Baseline** | 20.1 ± 1.9 | 17.7 ± 1.7 | 15.3 ± 1.2 | F_(3, 210)_ = 3.3, p = 0.02* | t = 2.1, p = 0.07 |
| **Week-2** | 17.9 ± 1.7 | 15.5 ± 1.5 | 15.2 ± 1.1 | Timepoint x arm: | Baseline-Week-8 |
| **Week-8** | 16.5 ± 1.7 | 16.3 ± 1.5 | 14.8 ± 1.5 | F_(6, 210)_ = 1.2, p = 0.3 | t = 2.5, p = 0.04* |
| **Week-32** | 16.2 ± 1.6 | 16.5 ± 1.6 | 14.2 ± 1.1 |  | Baseline-Week-32 |
|  |  |  |  |  | t = 2.9, p = 0.03* |
|  |  |  |  |  | Week-2-Week-8 |
|  |  |  |  |  | t = 0.3, p = 0.7 |
|  |  |  |  |  | Week-2–Week-32 |
|  |  |  |  |  | t = 0.7, p = 0.7 |
|  |  |  |  |  | Week-8-Week-32 |
|  |  |  |  |  | t = -0.4, p = 0.7 |
| **PANSS Total** |  |  |  |  | Timepoint: |
|  |  |  |  | Timepoint | Baseline-Week-2 |
| **Baseline** | 72.2 ± 6.0 | 60.9 ± 3.9 | 59.9 ± 2.7 | F_(3, 210)_ = 6.6, p = 0.0002* | t = 3.1, p = 0.005* |
| **Week-2** | 62.2 ± 4.5 | 54.6 ± 3.6 | 58.9 ± 2.7 | Timepoint x arm: | Baseline-Week-8 |
| **Week-8** | 60.5 ± 5.0 | 56.5 ± 3.4 | 57.2 ± 3.3 | F_(6, 210)_ = 1.6, p = 0.1 | t = 3.3, p = 0.003* |
| **Week-32** | 58.4 ± 4.9 | 56.7 ± 3.5 | 55.2 ± 2.9 |  | Baseline-Week-32 |
|  |  |  |  |  | t = 4.1, p = 0.0004* |
|  |  |  |  |  | Week-2-Week-8 |
|  |  |  |  |  | t = 0.2, p = 0.8 |
|  |  |  |  |  | Week-2–Week-32 |
|  |  |  |  |  | t = 1.0, p = 0.5 |
|  |  |  |  |  | Week-8-Week-32 |
|  |  |  |  |  | t = -0.7, p = 0.6 |
| **BNSS Total** |  |  |  |  | Timepoint: |
|  |  |  |  | Timepoint | Baseline-Week-2 |
| **Baseline** | 33.9 ± 3.9 | 27.8 ± 3.2 | 26.7 ± 3.2 | F_(3, 210)_ = 4.2, p = 0.006* | t = 1.6, p = 0.1 |
| **Week-2** | 30.7 ± 4.0 | 24.3 ± 3.2 | 26.0 ± 3.0 | Timepoint x arm: | Baseline-Week-8 |
| **Week-8** | 28.4 ± 4.3 | 24.8 ± 3.3 | 23.5 ± 2.8 | F_(6, 210)_ = 0.6, p = 0.7 | t = 2.7, p = 0.03* |
| **Week-32** | 25.6 ± 4.3 | 24.4 ± 3.4 | 22.9± 2.6 |  | Baseline-Week-32 |
|  |  |  |  |  | t = 3.4, p = 0.005* |
|  |  |  |  |  | Week-2-Week-8 |
|  |  |  |  |  | t = 1.0, p = 0.4 |
|  |  |  |  |  | Week-2–Week-32 |
|  |  |  |  |  | t = 1.7, p = 0.1 |
|  |  |  |  |  | Week-8-Week-32 |
|  |  |  |  |  | t = -0.7, p = 0.5 |
| **SNS Total^c^** |  |  |  | Timepoint |  |
| **Baseline** | 14.6 ± 1.5 | 15.8 ± 1.8 | 16.7 ± 1.2 | F_(3, 198)_ = 2.3, p = 0.08 |  |
| **Week-2** | 15.7 ± 1.8 | 14.4 ± 1.8 | 16.7 ± 1.1 | Timepoint x arm: |  |
| **Week-8** | 13.5 ± 1.7 | 13.9 ± 2.0 | 16.8 ± 1.5 | F_(6, 198)_ = 1.4, p = 0.2 |  |
| **Week-32** | 12.5 ± 1.7 | 14.3 ± 1.9 | 16.2 ± 1.3 |  |  |
| **BAG Total^d^** |  |  |  | Timepoint |  |
| **Baseline** | 3.1 ± 0.1 | 3.1 ± 0.1 | 3.1 ± 0.1 | F_(3, 192)_ = 1.7, p = 0.2 |  |
| **Week-2** | 3.3 ± 0.1 | 3.2 ± 0.1 | 3.1 ± 0.1 | Timepoint x arm: |  |
| **Week-8** | 3.1 ± 0.1 | 3.2 ± 0.1 | 3.1 ± 0.1 | F_(6, 192)_ = 1.1, p = 0.4 |  |
| **Week-32** | 3.0 ± 0.1 | 3.1 ± 0.1 | 3.1 ± 0.1 |  |  |
| **SOFAS Total** |  |  |  |  | Timepoint: |
|  |  |  |  | Timepoint | Baseline-Week-8 |
| **Baseline** | 44.4 ± 2.4 | 51.6 ± 2.5 | 47.9 ± 1.8 | F_(2, 140)_ = 12.9, p = 0.0006* | t = -3.9, p < 0.0001* |
| **Week-8** | 57.7 ± 3.8 | 54.2 ± 2.6 | 50.3 ± 2.1 | Timepoint x arm: | Baseline-Week-32 |
| **Week-32** | 58.1 ± 3.8 | 55.2 ± 3.0 | 51.2 ± 2.4 | F_(4, 140)_ = 4.2, p = 0.003* | t = -4.6, p = 0.0002* |
|  |  |  |  |  | Week-8-Week-32 |
|  |  |  |  |  | t = 0.7, p = 0.5 |
|  |  |  |  |  | Timepoint x arm: |
|  |  |  |  |  | Real rTMS real SCRT |
|  |  |  |  |  | Baseline-Week-8 |
|  |  |  |  |  | t = -5.2, p < 0.0001* |
|  |  |  |  |  | Baseline – Week-32 |
|  |  |  |  |  | t = -5.4, p < 0.0001* |
|  |  |  |  |  | Week-8-Week-32 |
|  |  |  |  |  | t = -0.1, p = 0.9 |
|  |  |  |  |  | sham rTMS real SCRT |
|  |  |  |  |  | Baseline-Week-8 |
|  |  |  |  |  | t = -1.2, p = 0.3 |
|  |  |  |  |  | Baseline-Week-32 |
|  |  |  |  |  | Week 8 – Week-32 |
|  |  |  |  |  | t = -1.8, p = 0.2 |
|  |  |  |  |  | Sham SCRT |
|  |  |  |  |  | Baseline-Week-8 |
|  |  |  |  |  | t = -1.1, p = 0.4 |
|  |  |  |  |  | Baseline-Week-32 |
|  |  |  |  |  | t = -1.6, p = 0.2 |
|  |  |  |  |  | Week-8-Week-32 |
|  |  |  |  |  | t = -0.4, p = 0.7 |
| **SLOF Total** |  |  |  |  | Timepoint: |
|  |  |  |  | Timepoint | Baseline-Week-8 |
| **Baseline** | 174.9 ± 5.8 | 183.7 ± 3.5 | 179.1 ± 3.9 | F_(2, 140)_ = 11.4, p = 0.0005* | t = -3.7, p = 0.0004* |
| **Week-8** | 184.8 ± 5.3 | 190.0 ± 3.1 | 183.0 ± 3.2 | Timepoint x arm: | Baseline-Week-32 |
| **Week-32** | 186.1 ± 4.8 | 190.4 ± 3.1 | 185.0 ± 3.3 | F_(4, 140)_ = 0.6, p = 0.7 | t = -4.5, p < 0.0001* |
|  |  |  |  |  | Week-8-Week-32 |
|  |  |  |  |  | t = 0.7, p = 0.5 |
| **PSP Total** |  |  |  |  | Timepoint: |
|  |  |  |  | Timepoint | Baseline-Week-8 |
| **Baseline** | 46.1 ± 2.7 | 51.4 ± 2.5 | 47.1 ± 2.0 | F_(2, 140)_ = 11.2, p = 0.00003* | t = -3.5, p = 0.0008* |
| **Week-8** | 57.2 ± 3.7 | 54.2 ± 2.7 | 49.4 ± 2.5 | Timepoint x arm: | Baseline-Week-32 |
| **Week-32** | 57.4 ± 3.7 | 54.3 ± 3.0 | 52.1 ± 2.5 | F_(4, 140)_ = 2.6, p = 0.04* | t = -4.5, p < 0.0001* |
|  |  |  |  |  | Week-8-Week-32 |
|  |  |  |  |  | t = -1.0, p = 0.3 |
|  |  |  |  |  | Timepoint x arm: |
|  |  |  |  |  | Real rTMS real SCRT |
|  |  |  |  |  | Baseline-Week-8 |
|  |  |  |  |  | t = -4.3, p = 0.0005* |
|  |  |  |  |  | Baseline-Week-32 |
|  |  |  |  |  | t = -4.4, p = 0.0005* |
|  |  |  |  |  | Week-8-Week-32 |
|  |  |  |  |  | t = -0.1, p = 0.9 |
|  |  |  |  |  | sham rTMS real SCRT |
|  |  |  |  |  | Baseline – Week-8 |
|  |  |  |  |  | t = -1.2, p = 0.3 |
|  |  |  |  |  | Baseline-Week-32 |
|  |  |  |  |  | t = -1.5, p = 0.3 |
|  |  |  |  |  | Week-8-Week-32 |
|  |  |  |  |  | t = -0.2, p = 0.9 |
|  |  |  |  |  | Sham SCRT |
|  |  |  |  |  | Baseline-Week-8 |
|  |  |  |  |  | t = -1.1, p = 0.4 |
|  |  |  |  |  | Baseline-Week-32 |
|  |  |  |  |  | t = -1.6, p = 0.2 |
|  |  |  |  |  | Week-8-Week-32 |
|  |  |  |  |  | t = -1.3, p = 0.3 |
| **UPSA-B Total** |  |  |  |  | Timepoint: |
| **Baseline** | 74.4 ± 6.0 | 76.3 ± 4.2 | 79.6 ± 2.2 | Timepoint | Baseline-Week-8 |
| **Week-8** | 80.5 ± 4.1 | 76.2 ± 4.3 | 78.7 ± 2.8 | F_(2, 140)_ = 3.2, p = 0.04* | t = -0.9, p = 0.3 |
| **Week-32** | 80.3 ± 4.2 | 78.2 ± 4.4 | 81.4 ± 2.7 | Timepoint x arm: | Baseline-Week-32 |
|  |  |  |  | F_(4, 140)_ = 1.3, p = 0.3 | t = -2.3, p < 0.07 |
|  |  |  |  |  | Week-8-Week-32 |
|  |  |  |  |  | t = 1.3, p = 0.3 |
| \| Note: rTMS: Repetitive Trnascranial Magnetic Stimulation; SCRT: Social Cognitive Remediation Therapy. ^a^one patient from real rTMS and real SCRT missing Mini-PONS; ^b^two patients from sham rTMS and real SCRT missing MSCEIT; ^c^one patient from real rTMS and real SCRT, one patient from sham rTMS and real SCRT, and two patients from sham SCRT missing SNS; ^d^two patients from real rTMS and real SCRT, two patients from sham rTMS and real SCRT, and two patients from sham SCRT missing BAG; * denotes a significant effect \| \| --- \| | | | | | |

*SI 10*: Adverse Events

We tested adverse events after each rTMS session, after the completion of 10 rTMS sessions (Week-2), and after the completion of 16 group SCRT sessions (Week-8). We used a binomial logistic regression model to test differences between treatment arms, and across all sessions, as well as their interaction of the daily reported adverse events (AE) in the rTMS treatment arms (Table 2a). No strong evidence that AE likelihood differed between treatment arms (b = -1.6, p-value = 0.2), across sessions (all b < 3.6, p-values > 0.09) or as a function of their interaction (all b < 2.2, all p-values > 0.09; please see Supplementary Table 7 for full details) was observed. Similarly, treatment arms did not differ in the number of reported AEs at Week-2 (X^2^ = 1.7, p-value = 0.2; Table 2b) or at Week-8 (X^2^ = 0.3, p-value = 0.6; Table 2c). Two serious adverse events (SAEs) occurred: one in the sham rTMS/real SCRT treatment arm and one in the sham SCRT treatment arm. In the sham rTMS/real SCRT treatment arm the SAE consisted of severe headache and vomiting 32 days after the last rTMS session. The SAE was classified as unrelated to the rTMS treatment and the patient returned for week-8 and week-32 assessments. In the sham SCRT treatment arm the SAE consistent of hospitalization due to psychotic relapse, driven by discontinuation of their prescribed antipsychotics and substance use. The patient attended one SCRT session and withdrew consent during the psychotic relapse. Likewise, no differences in comfort levels, engagement, anxiety and communication tendencies were observed between SCRT treatment arms (all X^2^ < 4.6, all p-values > 0.1; Supplementary Table 8).

*Supplementary Table* 7: Output for Binomial logistic regression for adverse events following rTMS sessions

| Predictor | Estimate (β) | Std. Error | z-value | p-value | Odds Ratio (OR) |
| --- | --- | --- | --- | --- | --- |
| (Intercept) | 3.88 | 2.22 | 1.75 | 0.08 | 48.43 |
| Treatment arm | -1.55 | 1.33 | -1.16 | 0.24 | 0.21 |
| Session 2 | -3.11 | 2.08 | -1.49 | 0.14 | 0.04 |
| Session 3 | -0.61 | 2.10 | -0.29 | 0.77 | 0.54 |
| Session 4 | -2.28 | 2.07 | -1.10 | 0.27 | 0.10 |
| Session 5 | 0.56 | 2.10 | 0.26 | 0.79 | 1.74 |
| Session 6 | -1.50 | 2.07 | -0.73 | 0.47 | 0.22 |
| Session 7 | -3.53 | 2.06 | -1.71 | 0.09 | 0.03 |
| Session 8 | -1.16 | 2.05 | -0.56 | 0.57 | 0.31 |
| Session 9 | -1.50 | 2.07 | -0.73 | 0.47 | 0.22 |
| Session 10 | -2.28 | 2.07 | -1.10 | 0.27 | 0.10 |
| Treatment arm:Session 2 | 2.16 | 1.28 | 1.69 | 0.09 | 8.68 |
| Treatment arm:Session 3 | 0.11 | 1.27 | 0.09 | 0.93 | 1.12 |
| Treatment arm:Session 4 | 1.34 | 1.26 | 1.06 | 0.29 | 3.81 |
| Treatment arm:Session 5 | -1.06 | 1.28 | -0.83 | 0.41 | 0.35 |
| Treatment arm:Session 6 | 0.56 | 1.26 | 0.44 | 0.66 | 1.74 |
| Treatment arm:Session 7 | 1.37 | 1.25 | 1.10 | 0.27 | 3.95 |
| Treatment arm:Session 8 | -0.20 | 1.26 | -0.16 | 0.87 | 0.82 |
| Treatment arm:Session 9 | 0.56 | 1.26 | 0.44 | 0.66 | 1.74 |
| Treatment arm:Session 10 | 1.34 | 1.26 | 1.06 | 0.29 | 3.81 |

*Supplementary Table* 8: Adverse events after 8-weeks of SCRT treatment for the ITT group

| **Question: I feel afraid** | | | | | | | | | |
| --- | --- | --- | --- | --- | --- | --- | --- | --- | --- |
| Group* | Strongly Agree (1) | Agree (2) | | Somewhat Agree (3) | Somewhat Disagree (4) | Disagree (5) | Strongly Disagree (6) | Mean | Kruskal-Wallis |
| real rTMS + real SCRT | 3 (18.8%) | 3 (18.8%) | | 4 (25.0%) | 3 (18.8%) | 1 (6.2%) | 2 (12.5%) | 3.12 |  |
| sham rTMS + real SCRT | 4 (23.5%) | 7 (41.2%) | | 3 (17.6%) | 2 (11.8%) | 1 (5.9%) | 0 (0.0%) | 2.35 | X^2^= 2.5;  p-value = 0.2 |
| Sham SCRT | 5 (22.7%) | 3 (13.6%) | | 6 (27.3%) | 5 (22.7%) | 3 (13.6%) | 0 (0.0%) | 2.91 |  |
| **Question: I don’t share anything personal about myself** | | | | | | | | | |
| real rTMS + real SCRT | 1 (6.2%) | | 4 (25.0%) | 1 (6.2%) | 7 (43.8%) | 1 (6.2%) | 2 (12.5%) | 3.56 |  |
| sham rTMS + real SCRT | 1 (5.9%) | | 7 (41.2%) | 4 (23.5%) | 3 (17.6%) | 2 (11.8%) | 0 (0.0%) | 2.88 | X^2^= 2.6;  p-value = 0.3 |
| Sham SCRT | 1 (4.5%) | | 5 (22.7%) | 3 (13.6%) | 11 (50.0%) | 2 (9.1%) | 0 (0.0%) | 3.36 |  |
| **Question: I withdraw as soon as possible** | | | | | | | | | |
| real rTMS + real SCRT | 3 (18.8%) | | 4 (25.0%) | 2 (12.5%) | 3 (18.8%) | 3 (18.8%) | 1 (6.2%) | 3.12 |  |
| sham rTMS + real SCRT | 3 (17.6%) | | 5 (29.4%) | 2 (11.8%) | 5 (29.4%) | 0 (0.0%) | 2 (11.8%) | 3 | X^2^= 1.4;  p-value = 0.5 |
| Sham SCRT | 2 (9.1%) | | 4 (18.2%) | 2 (9.1%) | 10 (45.5%) | 4 (18.2%) | 0 (0.0%) | 3.45 |  |
| **Question: I am unable to speak** | | | | | | | | | |
| real rTMS + real SCRT | 6 (37.5%) | | 6 (37.5%) | 2 (12.5%) | 2 (12.5%) | 0 (0.0%) | 0 (0.0%) | 2 |  |
| sham rTMS + real SCRT | 4 (23.5%) | | 8 (47.1%) | 2 (11.8%) | 3 (17.6%) | 0 (0.0%) | 0 (0.0%) | 2.24 | X^2^= 4.6;  p-value = 0.1 |
| Sham SCRT | 5 (22.7%) | | 3 (13.6%) | 6 (27.3%) | 7 (31.8%) | 1 (4.5%) | 0 (0.0%) | 2.82 |  |
| **Question: I become nervous** | | | | | | | | | |
| real rTMS + real SCRT | 1 (6.2%) | | 7 (43.8%) | 4 (25.0%) | 1 (6.2%) | 1 (6.2%) | 2 (12.5%) | 3 |  |
| sham rTMS + real SCRT | 2 (11.8%) | | 6 (35.3%) | 2 (11.8%) | 4 (23.5%) | 3 (17.6%) | 0 (0.0%) | 3 | X^2^= 4.3;  p-value = 0.1 |
| Sham SCRT | 1 (4.5%) | | 2 (9.1%) | 5 (22.7%) | 9 (40.9%) | 5 (22.7%) | 0 (0.0%) | 3.68 |  |
| Note: rTMS: Repetitive Trnascranial Magnetic Stimulation; SCRT: Social Cognitive Remediation Therapy. * 3/19 patients missing for real rTMS + real SCRT treatment arm; 9/26 patients missing for sham rTMS + real SCRT treatment arm; 6/28 patients missing for the sham SCRT teratment arm. | | | | | | | | | |

*Supplementary Figure* 1: Course of PSP until Week-8


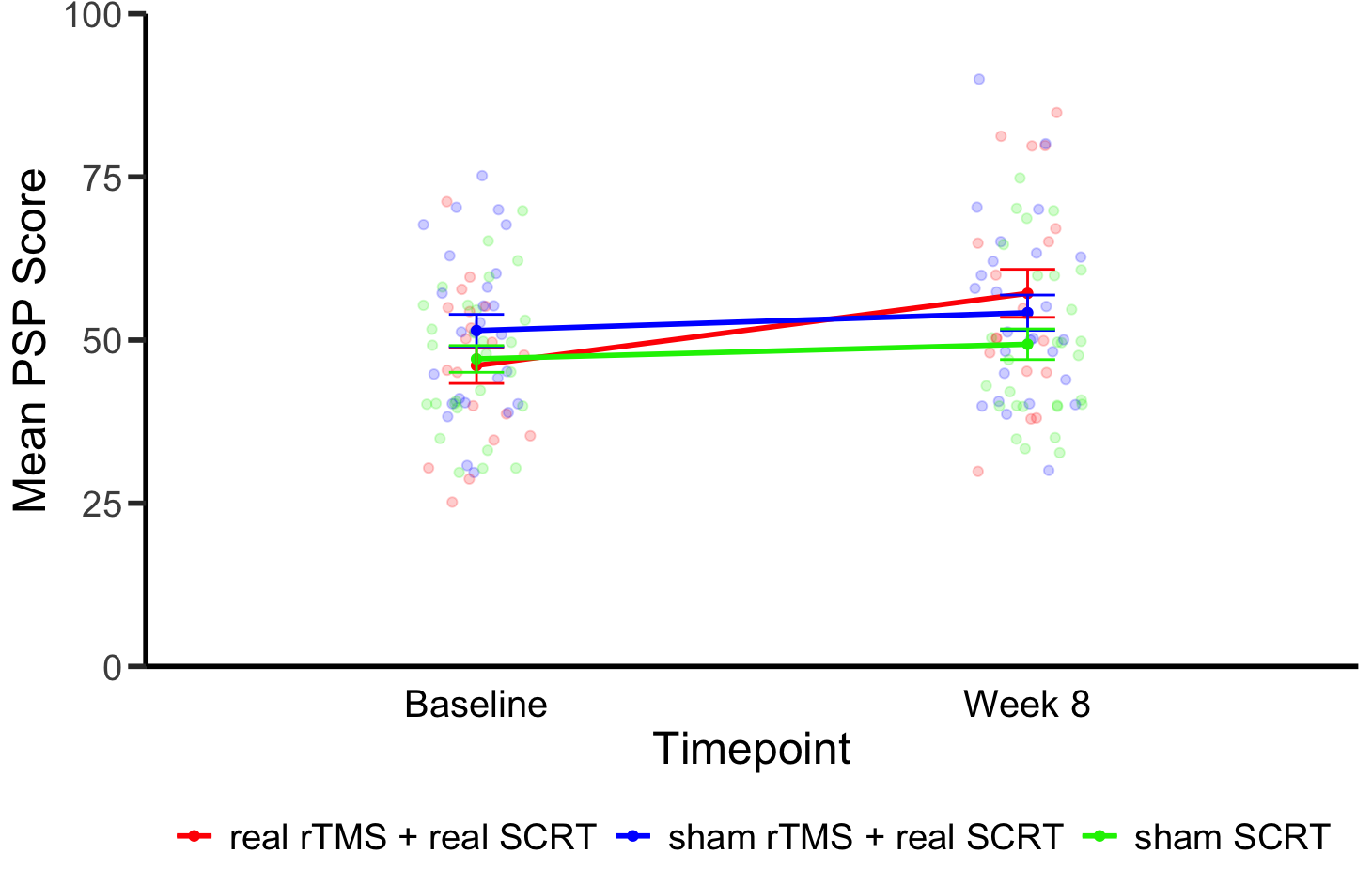


*Supplementary Figure* 2: Course of PSP until Week-32


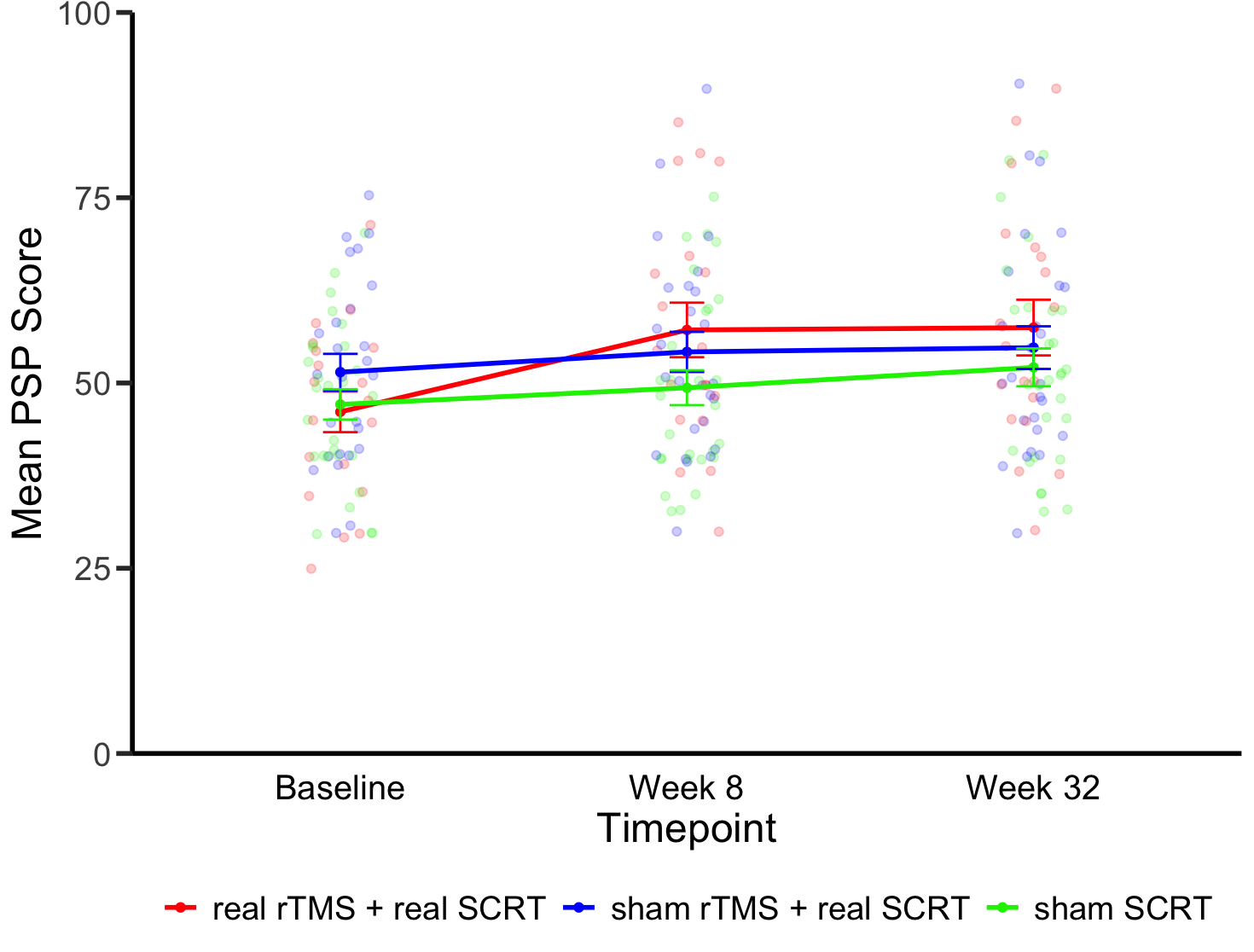


References

1. Chapellier V, Pavlidou A, Mueller DR, Walther S. Brain Stimulation and Group Therapy to Improve Gesture and Social Skills in Schizophrenia-The Study Protocol of a Randomized, Sham-Controlled, Three-Arm, Double-Blind Trial. *Front Psychiatry* 2022; **13:** 909703.

2. Roder V, Müller DR. *INT-integrated neurocognitive therapy for schizophrenia patients*. Springer International Publishing AG: Cham, Switzerland, 2015, x, 141-x, 141pp.

3. Vanbellingen T, Kersten B, Van Hemelrijk B, Van de Winckel A, Bertschi M, Müri R *et al.* Comprehensive assessment of gesture production: a new test of upper limb apraxia (TULIA). *European Journal of Neurology* 2010; **17**(1)**:** 59-66.

4. MEASURING SENSITIVITY TO NONVERBAL COMMUNICATION: THE PONS TEST*. 1979.

5. Mozaz M, Rothi LJG, Anderson JM, Crucian GP, Heilman KM. Postural knowledge of transitive pantomimes and intransitive gestures. *Journal of the International Neuropsychological Society* 2002; **8**(7)**:** 958-962.

6. Pavlidou A, Chapellier V, Maderthaner L, von Känel S, Walther S. Using dynamic point light display stimuli to assess gesture deficits in schizophrenia. *Schizophrenia Research: Cognition* 2022; **28:** 100240.

7. Mayer JD, Salovey P, Caruso DR. Mayer-Salovey-Caruso emotional intelligence test (MSCEIT) users manual. 2002.

8. Morosini PL, Magliano L, Brambilla L, Ugolini S, Pioli R. Development, reliability and acceptability of a new version of the DSM-IV Social and Occupational Functioning Assessment Scale (SOFAS) to assess routine social funtioning. *Acta Psychiatrica Scandinavica* 2000; **101**(4)**:** 323-329.

9. Hosmer Jr DW, Lemeshow S, Sturdivant RX. *Applied logistic regression*. John Wiley & Sons2013.

10. Mueller DR, Mueller-Szer R, Affentranger OB, Tschirren N, Wechsler FI. Changes in the experience and behaviour of schizophrenic patients in therapy groups improve functional skills and symptoms: RCT with 154 outpatients on cognitive remediation compared to standard treatment. *DGPPN Annual Congress*, vol. New Further Developments and Evaluation of Psychotherapy for People with Psychoses2023, pp 29.11.-22.12.2023.
